# Deep aerenchyma: a transformer-based pipeline for scalable phenotyping of rice root aerenchyma lacunae across environments

**DOI:** 10.1186/s13007-026-01546-1

**Published:** 2026-06-30

**Authors:** Hani Atef, Laura Fierro-Dominguez, Paula Andrea Lozano-Montaña, Sergi Navarro Sanz, Julie Bals, Benoît Clerget, Christophe Périn, Maria Camila Rebolledo, Romain Fernandez

**Affiliations:** 1 UMR AGAP Institut, INRAE, Montpellier, 34398 France; 2 CIRAD, UMR AGAP Institut, Montpellier, 34398 France; 3 International Center for Tropical Agriculture (CIAT), Cali, Colombia; 4 Barberi Faculty of Engineering, Design and Applied Sciences, Icesi University, Cali, Colombia

**Keywords:** Aerenchyma, Lacuna, Deep learning, Image segmentation, Rice, Root anatomy, Vision transformer, Phenotyping, High-throughput analysis

## Abstract

**Background:**

Quantification of rice root anatomical traits such as cortical aerenchyma lacunae is key to understanding rice adaptation to diverse water regimes and to support climate-smart breeding. Aerenchyma lacunae contributes to rice internal gas transport and influences methane emissions from flooded systems and can also limit rice water conductivity. It could be an interesting anatomical trait for breeding, however, large-scale anatomical phenotyping remains limited because manual analysis of root cross-sections is labor-intensive, subjective, and difficult to scale across heterogeneous imaging conditions. Existing pipelines often require parameter tuning and do not generalize well across environments.

**Results:**

We developed a deep learning pipeline based on a vision transformer architecture to automatically segment rice root cross-sections and quantify cortical aerenchyma lacunae. The model was trained on 1,760 annotated images collected across multiple countries, growth stages, cultivation systems, and experimental contexts, using a collaboratively defined annotation protocol. The final model achieved high segmentation accuracy, with mean intersection over union values exceeding 0.92 for cortical tissues and lacunae. Quantification of the lacuna-to-cortex ratio showed strong agreement with manual annotations, with a coefficient of determination of 0.98 on an independent test set. An independent expert review indicated that model predictions were at least as consistent as manual annotations and reduced large annotation inconsistencies. The pipeline is released as open-source software and includes an interactive online demonstrator, and is accompanied by an online test dataset to support testing and reproducibility. Application across six experimental use cases revealed reproducible differences in aerenchyma lacunae across genotypes, water regimes, environments, and developmental stages.

**Conclusions:**

This work provides a robust, scalable, and transferable tool for automated root anatomical phenotyping under heterogeneous experimental conditions. Transformer-based segmentation enables consistent and high-throughput quantification of lacunae, facilitating integration of these anatomical traits into breeding, physiological studies, and climate-smart crop improvement programs.

**Supplementary Information:**

The online version contains supplementary material available at https://doi.org/10.1186/s13007-026-01546-1.

## Introduction

Cortical root traits play a central role in climate adaptation and resilience in Poaceae [12], highlighting the need for phenotyping tools that enable their effective incorporation into breeding programs. Quantifying anatomical traits in roots is instrumental for modelling plant water uptake, gas transport, and adaptation to environmental constraints [2, 15]. In roots, aerenchyma is a cortical tissue characterized by enlarged gas spaces, the aerenchyma lacunae, exceeding typical intercellular voids, and playing a dual role in rice [21]. Presence of aerenchyma lacunae supports oxygen and methane transport under flooded conditions [10] or can reduce root metabolic cost under drought [4]. However, its presence can also affect the radial water conductivity under non-flooded aerobic conditions [20]. As a result, accurate and reproducible measurement of aerenchyma lacunae and cortex area is essential for studies aiming to identify genotypic variation in water use efficiency, methane emissions and tolerance to anoxic conditions.

Several tools have been developed to extract anatomical traits from root cross-sections. Early semi-automatic pipelines such as RootScan [3] and PHIV-RootCell [14] rely on rule-based image processing implemented in ImageJ, requiring user-guided segmentation steps and threshold tuning. These methods enable the quantification of features such as stele diameter, cortex width, and vessel number, and can indirectly inform estimates of aerenchyma lacunae proportion. However, they are sensitive to image variability and remain dependent on manual supervision, which limits their throughput and reproducibility across developmental stages, varieties, and culture conditions. RootScan was developed and validated primarily using crown roots from young plants of maize and, to a lesser extent, rice and beans (25–28 days after sowing) grown under relatively controlled and field conditions. By contrast, PHIV-Rootcell was designed for analysing very young rice seedlings (6 days after sowing) that are grown under highly controlled conditions on a Murashige and Skoog medium, focusing on the root tip cross-section of developing radicles. RootAnalyzer [7] further extends automated anatomical analysis to a later developmental stage, having been validated on primary roots of wheat (*Triticum aestivum* L.) genotypes grown under contrasting water regimes (well-watered and drought), with cross-sections obtained approximately 4 cm from the root tip at 50–56 days after sowing. While these earlier tools advanced the field, segmentation of root cortex aerenchyma lacunae of rice across diverse environments and varieties remains challenging and requires approaches that ensure robustness to heterogeneous data sources and observation conditions.

In recent years, numerous deep plant phenomics tools have emerged [23], and root anatomics make no exception. RootAnalyzer [7], which applies contour-based segmentation with fixed pipelines, and DL-RootAnatomy [24], which uses object detection networks (Faster R-CNN) to identify anatomical regions, are representative examples. However, while these approaches reduce manual intervention, they are trained on controlled laboratory images, and their robustness under more variable field-derived conditions remains uncertain. Furthermore, none of the existing tools was specifically designed to segment the highly variable cortical lacunae. For instance, RootAnalyzer only provides a rudimentary classification of large cortical air spaces, and DL-RootAnatomy does not address them explicitly. As a result, many laboratories still rely primarily on manual or semi-manual analysis of root cross-sections, especially for cortical aerenchyma. This continued dependence on manual analysis creates a major bottleneck for large-scale experiments, motivating the development of automated methods that remain robust across heterogeneous datasets. But classical thresholding and fixed rule-based segmentation remain insufficient to quantify cortical aerenchyma lacuna in our imaging context. In these epifluorescence cross-sections, lacunas are not revealed by a specific stain, and both living cortical cells and air spaces appear as cavity-like regions bounded by fluorescent walls. As a result, reliable identification depends not on intensity alone, but on higher-order morphological cues such as wall waviness, partial lysis, coalescence of neighbouring cavities, radial elongation, and consistency with the surrounding cortical organization. These cues are further perturbed by variability in contrast, blur, and observation conditions across experiments, making robust hand-crafted pipelines difficult to generalize. This is precisely the type of visual ambiguity where data-driven AI approaches are particularly relevant.

These tools have not yet been applied to distinguish anatomical differences across genotypes, treatments, or developmental stages in practice, limiting their applicability to support ongoing breeding efforts. Recent efforts in acquisition systems have pushed anatomical phenotyping possibilities toward higher throughput using custom-built platforms such as the Rapid Anatomics Tool (RAT) [11], including an efficient and scalable mechanical sectioning design, combined with a semi-automatic analysis pipeline. High-resolution 3D imaging approaches, such as Laser Ablation Tomography (LAT), provide access to volumetric anatomy [16], but they rely on specialized instrumentation and dedicated 3D analysis workflows, which can limit their routine adoption across laboratories and large phenotyping programs.

Together, these tools highlight a gap in AI-driven plant research: the lack of automated methods fitting with the standard laboratory imaging and computing environment, capable of quantifying aerenchyma lacunae formation in mature zones of roots, where cortical air spaces are highly heterogeneous and influenced by genotype, environment, and developmental stage. In this work, we present a robust and scalable pipeline for anatomical segmentation of rice root cross-sections using transformer-based deep learning methods. Our method focuses specifically on the rice cortex and cortical aerenchyma lacunae. It is trained on a multi-environment, field-derived dataset of nearly 1800 curated images and requires no parameter tuning. Built on the SegFormer architecture [25], our model accurately segments the cortex and lacunae, achieving expert-level performance across heterogeneous acquisition conditions, as confirmed through an independent expert review. The pipeline is fully open-source, includes a web-based interface for non-specialist users, and is routinely used in ongoing phenotyping campaigns. Finally, we demonstrated that this tool differentiates rice cortical aerenchyma lacunae across different water treatments, genotypes, growth stages, and growth environments (from hydroponics to field). By providing a fast, reproducible, and field-validated method for quantifying aerenchyma lacunae, this work contributes a practical tool for large-scale anatomical phenotyping supporting rice research for climate-smart breeding.

## Methods

### Plant material and imaging

**Fig. 1:**
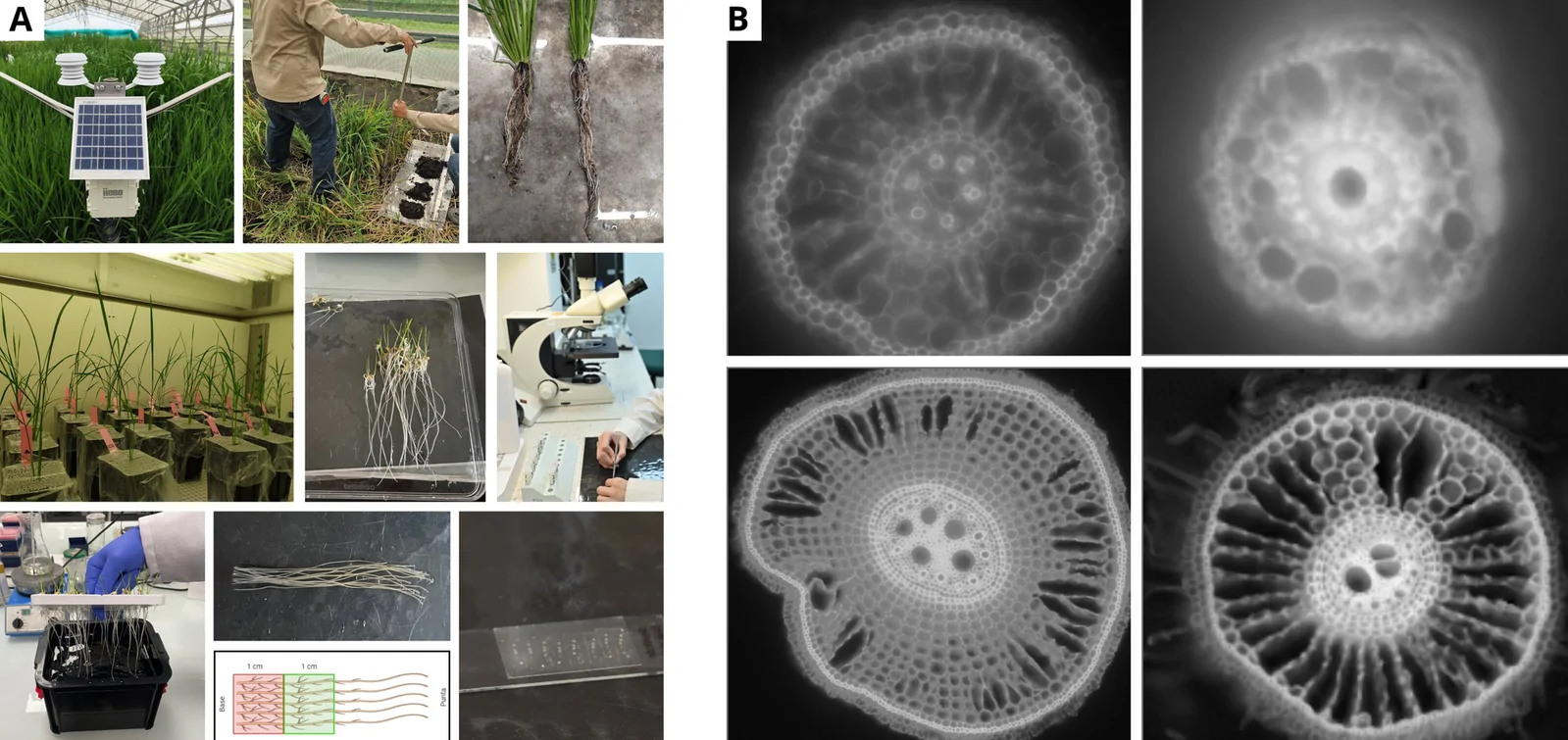
Culture environments, imaging setups, and variability of rice root cross-section images. **A** visual overview of growth conditions, harvesting procedures, and imaging setups from which the root cross-section images were obtained from the six use cases included in the dataset, illustrating the diversity of conditions. **B** Representative rice root cross-sections images acquired with epifluorescence microscopy, illustrating the diversity of anatomical appearances encountered across experiments, including different degrees of aerenchyma development. Early-stage examples show small, irregular lacunae that remain close to the scale of individual cortical cells (upper line), whereas late-stage examples (bottom line) show larger, radially elongated, and partially coalescent air spaces extending across several cortical cell layers.

Root cross-sections were obtained from multiple experiments conducted in different countries (Colombia and France) and environments (hydroponics, petri dishes, pots, and field). See Fig. 1; Table 1. Root segments were collected from either seminal roots (hydroponic) or crown roots (soil or pot) and excised at either 1, 2, or 3 centimeters from the root tip (hydroponic) or 1 cm from the root base (soil or pot). The global dataset (across use-cases) corresponds to different plant developmental stages and root maturity levels (and lengths). Thus the dataset includes a representative variability of section position relative to the root tip, and hence, a continuum of aerenchyma lacunae maturation levels.

**Table 1:** Summary of the use-cases providing images and annotations, including characteristics of their experiments

Use-case code	Country	Rice varieties	Growth stage	Growing conditions	Research group	Experimenter
UC1-JUL	France	Mutants from a background of Kitaake	5 leaves	Hydroponic in a growth chamber	CIRAD/DARS	Julie Bals
UC2-HYDRO	Colombia	Panel of 20 Indica, Japonica, Aus, O. rufipogon, and Tropical japonica	6–7 leaves	Hydroponic in a glass greenhouse	CIAT	Paula Lozano
UC3-SHELTER	Colombia	Panel of 19 *Indica*,* Japonica*,* Aus*, *Oryza rufipogon*, and *Tropical japonica*	6–7 leaves and panicle initiation	Rain-out shelter with soil, flooded / not flooded	CIAT	Paula Lozano
UC4-MUT	France	Mutants from the Kitaake genotypes	5 leaves ; Sect. 1 cm from root base	Growth chamber, pots with substrate	CIRAD/ PHENOMEN	Domitille Tur
UC5-VARIETIES	France	Panel of 10 Indica and japonica tropical	5 leaves	Greenhouse, pots with soil, flooded / not flooded	CIRAD/ PHENOMEN	Justin Savajols
UC6-LFD	France	IR64 (Indica) and Primavera (japonica)	5 leaves	Greenhouse, pots with soil, flooded / not flooded	CIRAD/ PHENOMEN	Laura Fierro Dominguez

The segments were embedded in 5% agar and sectioned transversely using a vibratome, producing uniform histological slices for anatomical analysis. Imaging was performed using epifluorescence microscopy under controlled acquisition settings. Illumination intensity was maintained at 85% of maximal lamp power, and images were captured at magnifications ranging from 10x to 20x, depending on root diameter. All images were acquired as 8-bit grayscale TIFF files with sufficient resolution to visualize cortical tissues, the endodermis, cell walls, and lacunae.

For autofluorescence analyses in France, images were collected at room temperature on Ni-E microscope (Nikon) equipped with a Sola SE UV365 lamp, a Nikon 10X Plan Apo λ 0.45 NA and/or Nikon 20X Plan Apo λ 0.75 NA and a DAPI filter (excitation [Ex] 330/50 nm, dichroic mirror [DM] 400 nm, emission [Em] 420/50). Images were acquired with DS-Ri2 camera (Nikon) controlled with NIS-Elements software (Nikon) with the following camera settings: exposure 200ms, Gain 5x, binning 1.0 × 1.0, Brightness 0, Hue 0 and Saturation 0.

For autofluorescence analyses at CIAT, Colombia, images were collected at room temperature on a Leica Microsystems CMS GmbH DMi8 manual microscope equipped with a TL LED lamp (S/N 390183, LED U module), a LED_365 Blue-white filter (excitation [Ex] 340–380 nm, dichroic mirror [DC] 455 nm, emission [Em] 450–490 nm), and an integrated DFC310 FX camera (default camera). Images were acquired and controlled with LAS X software version 3.7.1.21655 (Leica) with the following camera settings: exposure commonly 400–1050 ms (rarely 350–2500 ms), gain 2.0, binning 1 × 1 (1392 × 1040), Brightness 0, Hue 0 and Saturation 0.

The resulting dataset comprised 2,498 raw microscope images prior to curation, distributed across several acquisition batches with variable image quality (Fig. 1). Detailed metadata (site, genotype, treatment, acquisition date, magnification) were kept to assess robustness across varying conditions.

### Image quality control and dataset curation

**Fig. 2:**
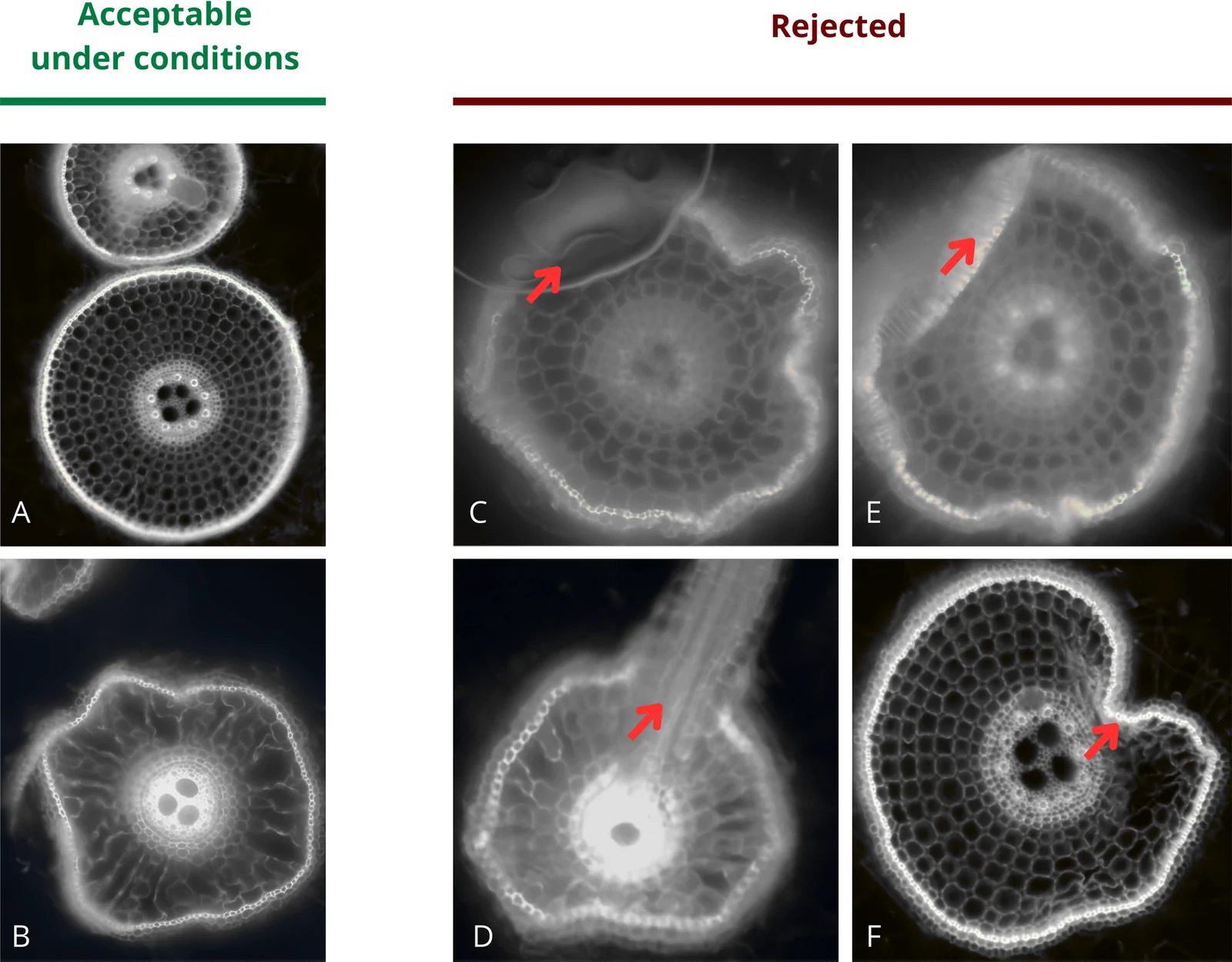
Representative examples of curated rice root cross-section images. **A** a correct root cross-section, surrounded by an additional object, that will be automatically removed during processing after cortex segmentation by selecting the bigger cortex region. **B** limit of circularity for accepting a cross-section, to foster an acceptable level of biological meaning in the measurements. **C-F** cross-sections presenting at least one important defect, and therefore rejected during manual screening (**C**: water drop, **D**: presence of a lateral root, **E**: the slice is cut, **F**: the slice is damaged with a large intrusion of sclerenchyme, probably by manipulation).

To exclude damaged or low-quality images, we developed a custom Python graphical interface to enable rapid visual inspection and triage. Images were manually flagged for removal based on the following criteria: blur or defocus artifacts on a large portion of the slice (Fig. 2C), incomplete or truncated root sections, or additional structures (Fig. 2A, C, D and E), topological artifacts obstructing the cortex (Fig. 2D), and extreme shape deformation or anatomical ambiguity preventing reliable annotation (Fig. 2D and F). These categories cover many sources of image defects, including the presence of large water droplets preventing visualization of part of the slice; one or more lateral roots inducing supporting tissue that alters the local aerenchyma lacunae distribution; and large concavities in the slices, indicative of damaged roots and unreliable measurements of cortex surface. After the manual flagging step, the tool generated a full audit report in CSV format, which was used to give feedback to the image providers to enable discussion and improvement. After this curation step, 1,760 images (70.5% of the original dataset) remained for model development.

### Target definition

#### Aerenchyma lacunae genesis and visual phenotype

Our image analysis objective is the identification and quantification of cortical aerenchyma lacunae, the air-filled cavities that develop within the cortex. The lacuna considered here are lysigenous, arising from programmed cell death and the progressive lysis and collapse of specific cortical cells, which generate interconnected air spaces that facilitate internal aeration under hypoxic stress. Aerenchyma lacunae development is gradual, and our dataset captures a continuum of maturation stages (Fig. 1).

**Fig. 3:**
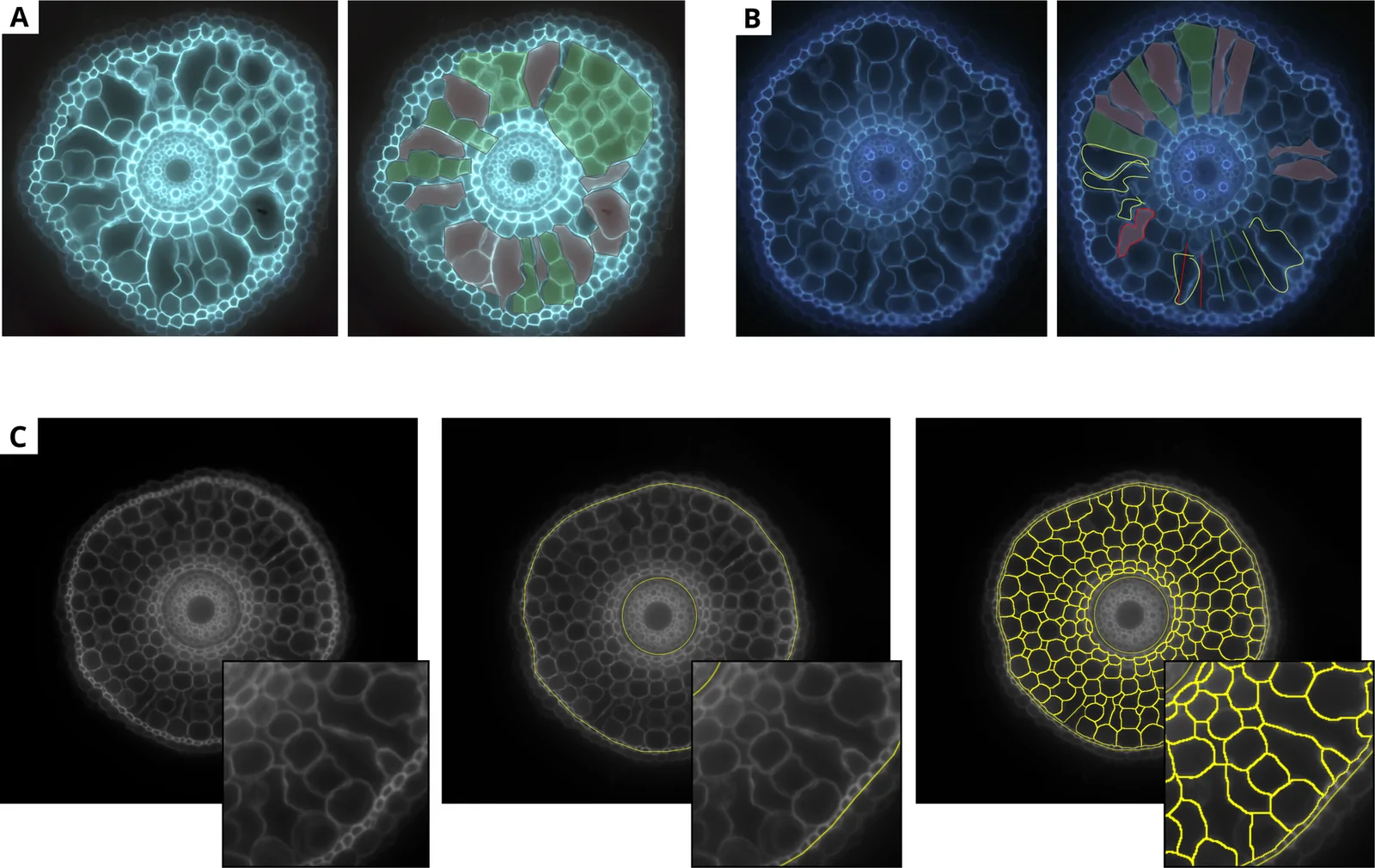
Annotation guide and manual annotation workflow for cortical aerenchyma. **A**, **B** Representative excerpts from the annotation guide used to harmonize difficult annotation decisions before large-scale manual annotation. In each example, left image is raw and right image is annotated. The candidate regions were discussed collectively to decide whether they should be labelled as aerenchyma lacunae or not on the basis of morphological criteria. Colored overlays indicate the proposed class assignment, while the superimposed contours and comments illustrate the consensus-building discussion among annotators on a shared document. These examples highlight ambiguous cases such as partially lysed cortical cells, merged lacunae, and irregular cavities. **C** Step-by-step manual annotation workflow. Left: original image. Middle: manual delineation of the inner and outer cortical boundaries. Right: manual identification of aerenchyma regions on the watershed-derived partition using the “Click Aerenchyma” tool.

In our epifluorescence cross-sections, aerenchyma lacunae is not revealed by a specific stain; instead, images mainly show general cell-wall autofluorescence, so both living cells and air spaces appear as “holes” bounded by fluorescent walls. Consequently, aerenchyma lacunae identification relies on a combination of morphological cues rather than a dedicated intensity signature. In early stages, incipient aerenchyma lacunae can be subtle: healthy cortical cells are typically polygonal and tightly packed, whereas the first lysing cells exhibit tortuous or wavy walls, local wall discontinuities, and partially emptied lumens (Fig. 3A, B). With progression, adjacent lacunae expand and coalesce into larger cavities. At advanced stages, these cavities are often larger than individual cells and become elongated along the root radius (centre-to-periphery), consistent with the fact that mature aerenchyma lacunae can span what would normally correspond to multiple radial cortical cell layers. Figure 1B illustrates representative early and late aerenchyma lacunae morphologies, ranging from small irregular lacunae to large continuous air channels traversing the cortex.

#### Morphological variability and annotation challenges

The morphogenetic trajectory creates practical challenges for annotation. Early-stage aerenchyma lacune may be difficult to separate from normal cell lumens. In contrast, late-stage aerenchyma lacunae may blur conventional notions of “cell boundaries” because collapsing walls and coalescence can make neighbouring compartments appear merged. To a human observer, advanced aerenchyma lacunae may even appear as if an entire radial file of cortical cells is missing, with surrounding cells seemingly “fusing” across the cavity.

Capturing this morphological variability consistently in the annotations was essential, because segmentation choices directly affect the quantitative traits extracted from the images, especially the proportion of aerenchyma within the cortex. To reduce inter-annotator variability, we developed a detailed annotation guide, with representative examples shown in Fig. 3A, B. In this study, a cortical aerenchyma lacuna was defined as a cavity located within the root cortex and resulting from lysigenous cell lysis. In epifluorescence images, lacunae were identified using morphological criteria rather than a specific intensity signature. These criteria included disruption of the normal polygonal organization of cortical cells, tortuous or discontinuous cell walls, partially emptied lumens, local coalescence of neighbouring cavities, radial elongation, and unconsistency with the surrounding cortical pattern. Small regular lumens of intact cortical cells and xylem vessels located in the stele were not annotated as aerenchyma lacunae. The annotators’ guide combined these explicit decision rules with visual examples of challenging cases, including waviness of cell walls, partial wall lysis, merged intercellular spaces, and radial elongation of mature lacunae. Figure 3C shows the corresponding manual annotation workflow used to generate the training masks. This design was motivated by evidence that annotation errors in biomedical image analysis are often driven less by annotator skill than by ambiguous or underspecified labelling instructions, and that concrete visual examples can substantially improve consistency and accuracy [19]. By integrating these best practices into our protocol, we aimed to maximize inter-annotator agreement and produce higher-quality manual reference annotations to be used as ground truth for training and evaluation. However, despite the shared guide, some inter-annotator variability remained expected in difficult cases, especially for young or weakly differentiated aerenchyma, merged cavities, blurred cortical boundaries, or images with low contrast, where most disagreements appeared regarding the inclusion of small ambiguous lacunae.

### Manual annotation workflow

The annotation team included both co-authors and non-co-author contributors with complementary experience in rice microscopy, plant anatomy, and laboratory image analysis. Ground truth masks were generated through a semi-automated Fiji/ImageJ annotation workflow using the target definition guide and workflow tutorial. The workflow was divided into a sequence of manual actions over all images, followed by a sequence of automated actions, in order to streamline annotations across a large number of images. Overall, the manual operations required approximately three minutes per slice, resulting in an approximately 90-hour annotation effort distributed across multiple operators and multiple laboratories.

Starting from the raw image (Fig. 3C, left), annotators first delineated the inner and outer cortical boundaries (Fig. 3C, middle), using custom macros based on polygon tracing. A watershed-based cell segmentation step (Fig. 3C, right) was then applied to highlight internal structures, which helped identify air-filled lacunae and cytoplasm within the cortex. Using the dedicated Click aerenchyma tool, annotators manually identified the segmented structures corresponding to aerenchyma lacunae, following the annotation guide. A binary morphological closing operation was then applied to fuse potential aerenchyma lacunae subregions resulting from oversegmentation errors. The workflow included image verification steps to help annotators flag images that required re-annotation. After the final step, the workflow produced segmented images comprising three classes: background and central cylinder; cortical lacunae; and cortex-endodermis region. These masks obtained from manual reference annotation were exported as binary TIFF format and served as ground truth for model training.

### Data preparation and augmentation

To facilitate an efficient and deployable pipeline, we downscaled all images from the original image size to a fixed target size of 512 × 341 pixels. While all the image of the training dataset have an aspect ratio from 1,3 to 1,5 (“landscape format”), the pipeline pads image before resizing if the aspect ratio of the input image differs of more than 20% of this aspect ratio range, enabling to process a broader diversity of input images.

While this downscaling inevitably sacrifices some fine detail visible to human observers, previous work has shown that moderate image downsampling can maintain or improve network accuracy [9] by providing more contextual information relative to each pixel, because extremely high resolutions contain detail beyond the effective receptive field of the network which can lead to fragmented predictions, whereas a lower resolution can better align with the object size and network context window. In this context, we opted for whole-image downscaling rather than dividing each image into high-resolution patches with a tiling-and-stitching approach. The chosen 512 × 341 resolution thus represents a pragmatic balance between retaining sufficient visual information and achieving fast, CPU-friendly processing. This uniform reduction in size dramatically lowers memory usage and inference time, enabling the use of more advanced models or deployment on lower-capability hardware. As a result, the final model can process images at roughly one frame per second on a standard CPU, which enabled us to release a free, real-time demo via Hugging Face (Fernandez [1]) running on non-specialized hardware and publicly accessible without subscription.

We separated the test set at the start to avoid data leakage and built it using a random sampling of 236 images, spanning all image providers. The remaining dataset was used as a training set, which was augmented before training and hyper-parameters fine-tuning. The 1,760 manually annotated images from two different laboratories with different sampling preparation methods and spanning six heterogeneous use cases are aggregated with the aim to capture a broader range of microscope image qualities but also the differences generated by the developmental, varietal, environmental stage, and image acquisition pipelines that are typically represented in more controlled root anatomy datasets. However, this dataset size can be considered as average-sized or small-sized for deep learning, depending on the neural network architecture depth, and the number of trainable weights. To overcome this, we used an augmentation strategy. Because the root cross-sections exhibit radial symmetry, we applied extensive rotation-based data augmentation. Each original image-mask pair was augmented with 24 rotated copies at regular 15° intervals (covering rotations from 0° to 345° in 15° increments). These rotations are biologically valid for cross-sections, which have no inherent “upright” orientation, thereby increasing training samples without introducing biologically implausible features. Additionally, we applied horizontal and vertical flips, as well as small random translations (± 10 pixels). This multi-faceted augmentation strategy expanded the training dataset to several hundred thousands augmented samples, providing an extensive set of examples to improve the model’s robustness.

### SegFormer architecture and training procedure

We selected a vision transformer architecture and adapted it by designing the segmentation head to produce two output channels corresponding to the cortex-endodermis region (CE) and the aerenchyma lacunae class (AR). We used the SegFormer 2D semantic segmentation framework [25], which combines a hierarchical Mix Transformer (MiT) encoder with a lightweight all-MLP decoder. SegFormer was originally introduced and benchmarked on natural-image segmentation datasets such as ADE20K, while its encoders are commonly initialised using ImageNet-1 K pre-training before fine-tuning. Its design is well-suited to our task because it produces multi-scale feature maps that capture both global root organisation (cortex outline and tissue interfaces) and small-to-medium structures with high morphological variability (individual lacunae). In addition, SegFormer does not use explicit positional encodings, avoiding the need to interpolate positional embeddings when inference resolution differs from training. This is advantageous in our workflow, where images are systematically resized for computational tractability and CPU-friendly deployment.

In practical terms, the model first transforms the input root cross-section image into a hierarchy of feature maps capturing information at multiple spatial scales, from global tissue organization to local cavity morphology. These multi-scale representations are then combined by a lightweight decoder to generate pixel-wise predictions for the two target classes, namely the cortex-endodermis region and the lacunae. These predictions are then converted into binary segmentation masks, and the lacuna mask is further constrained by the cortex mask, such that lacuna regions predicted outside the segmented cortex are discarded as anatomically invalid. The pipeline therefore converts a raw microscopy image into anatomically constrained segmentation masks, without the need for hand-crafted feature extraction or manually tuned image-processing rules.

SegFormer is available in multiple variants, based on a family of backbones of increasing capacity from B0 to B5. In this study, backbone selection was guided by a preliminary screening of B0 to B4 focused on practical deployability. We considered both the published accuracy-complexity trade-off of the SegFormer family [25] and the practical inference time observed in our settings, both online (the Hugging Face space used with free access, offering a single CPU) and offline (on a recent laptop with a i7 processing unit with 16 cores, and 64 GB memory). The computation time per image of the pipeline with the B4 backbone was measured to approximately 2.5 s online and 0.08 s per image offline. On this basis, SegFormer-B4 was selected for final training and deployment, as it provided an appropriate compromise between expected segmentation performance and the observed computational time for routine use in both cases.

The models were trained in PyTorch on the Jean Zay supercomputing facility (GENCI, France) using NVIDIA H100 GPUs. Unless otherwise stated, training hyper-parameters were: batch size 8, input size 512×512 (after padding), AdamW optimiser, learning rate 1×10⁻⁴ with cosine decay, and weight decay 0.01. We used a combined loss function,

1 $$ \begin{aligned} {\text{Loss = }} & \alpha {\text{ }} \times {\text{ Dice Loss + }}\left( {{\text{1 - }}\alpha } \right) \\ & \times {\text{ Binary cross - entropy loss}} \\ \end{aligned} $$

with α ∈ {1, 0.9, 0.7, 0.5} to assess robustness to loss weighting. Models were trained for up to 100 epochs with early stopping based on validation performance. Training logs were recorded using TensorBoard, with reported metrics including standard metrics such as intersection over union [22].

### Evaluation of the pipeline

#### Automatic evaluation

Training logs were recorded using TensorBoard throughout all experiments. Model performance was first quantified by tracking both the evolution of global loss and intersection over union (IoU) for cortex–endodermis, and lacunae classes. After training, we assessed visual performance on a test set of images unseen by the model, drawn from all acquisition sites and experimental conditions, and summarized the results by reporting epoch-wise IoU on the training and validation sets, and final lacuna-to-cortex ratio R² for each use case (hydroponics, shelter, field) and for the full test set.

#### Independent expert review

We complemented these automatic metrics with an independent expert review to investigate the largest discrepancies and draw conclusions about the ability of the model to adhere to the annotation criteria. First, we ranked all test images according to a disagreement score between manual and model masks (maximizing 1 - IoU). From this ranking, we randomly sampled 23 images among those with the largest disagreements, corresponding to cases where at least one of the two segmentations (manual or model) was substantially incorrect. For each selected image, we identified the segmented aerenchyma lacunae components contributing to the discrepancy (connected pixel regions of lacunae that differed between masks) and presented them to the evaluators. Two senior rice experts, Christophe Périn and Benoît Clerget, who were not involved in the initial annotation process, independently inspected the raw images together with the manual and model segmentations. These two experts, co-authors of this study, have approximately 20 and 15 years of experience working on rice, respectively. For every discrepant aerenchyma lacunae component, they assigned one of three labels: (i) error in the manual annotation, (ii) error in the model prediction, or (iii) undefined. The final counts are reported in Table 2.

### Visibility, accessibility, and reproducibility

The multi-environment multi-site dataset used for training is available upon reasonable request, subject to data-sharing agreements.

To ensure reproducibility by the community, all code used for preprocessing and training is released under an open-source licence on GitHub, tagged v1.0.2, and archived with a Zenodo DOI [1].

For users who want to run the pipeline on images, two convenient options are provided with the HuggingFace space (Fernandez [1]): (i) an interactive web demonstrator deployed as Hugging Face Space, which requires no installation and includes a subset of the test dataset; or (ii) a local version of the pipeline available from the same repository, which can be cloned from the tab “Files” of the space, installed following the instructions provided in the README.md, by installing the libraries defined by the requirements, and executing the run_locally.py script on any machine equipped with a GPU and a standard Python environment.

The online demonstrator allows non-specialists to upload a batch of images stored in a ZIP archive and obtain predictions in a limited time (approximately 2 s per image) without any local installation.

The offline script can process images at a significantly faster throughput (0,08 s per image, as measured on a recent lenovo laptop with a nvidia GPU).

## Results

### Performance of SegFormer-B4 for aerenchyma lacunae and cortex segmentation

**Fig. 4:**
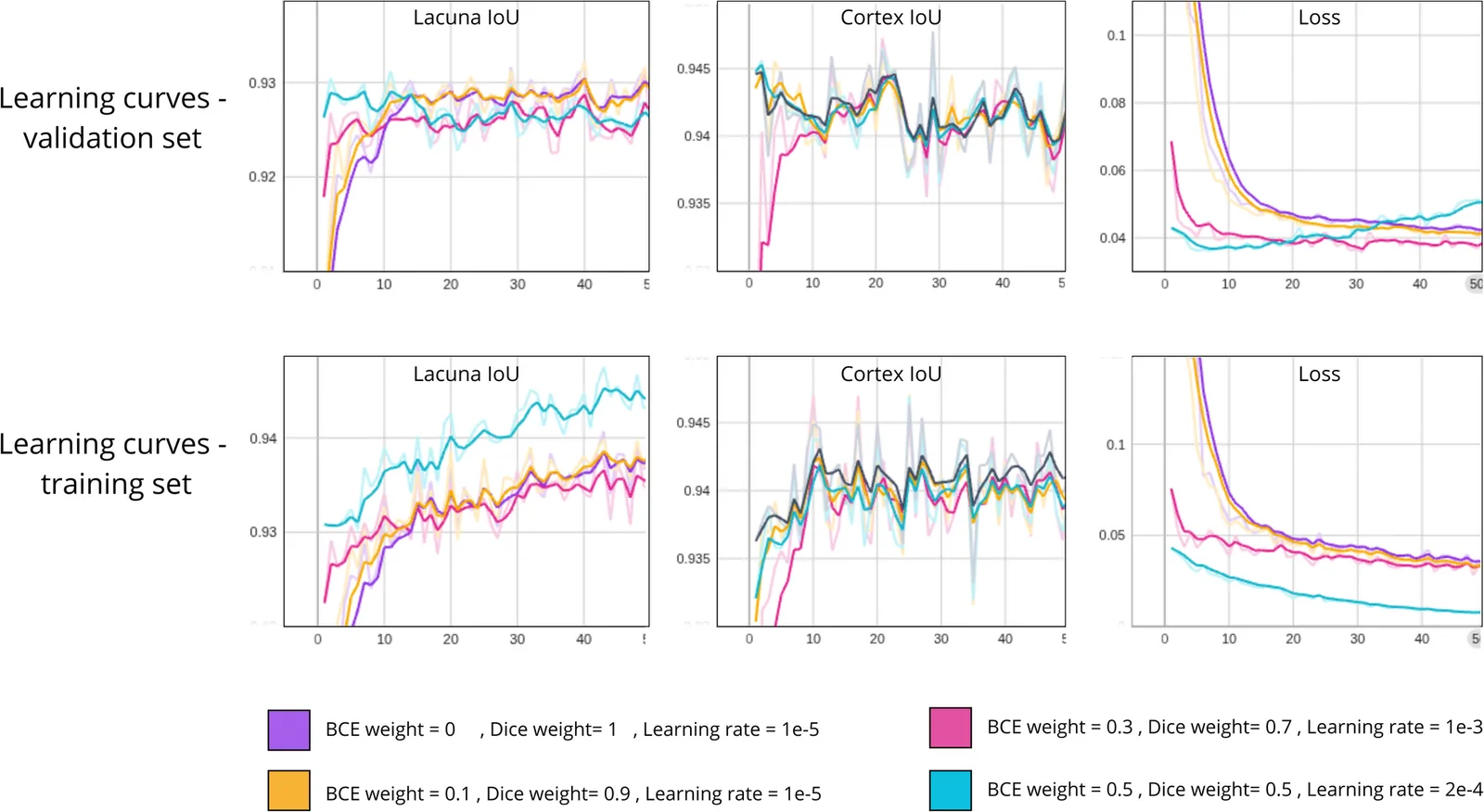
Training and validation performance (y axis) versus training epochs (x axis) across loss configurations for cortical and lacuna segmentation. Training and validation curves are shown for both cortex segmentation and aerenchyma lacunae segmentation, illustrating convergence behaviour under different loss formulations.

Across most hyper-parameters configurations, the SegFormer-B4 model converged rapidly and consistently, reaching high intersection over union values on the validation set (Fig. 4, upper panel) after a few epochs. Across the four best configurations, the validation IoU reached 0.90 after 3 epochs, 0.92 after 5 epochs, and its best value after 10 epochs. Final mean validation IoU values ranged from 0.925 to 0.929 for lacunae and from 0.943 to 0.944 for cortical tissues (CE), with minimal variability across loss-weighting settings, indicating stable optimization and consistent performance.

In the training set curves (Fig. 4, bottom panel), the loss profiles (right panel) showed a smooth decline during the first 10 epochs and mostly plateaued thereafter, in conjunction with a change in the validation loss trend (upper-right plot) that indicates the start of training set overfitting and a reduction in generalization. Some variations were identified in the exact training curve trends, while final models showed very small IoU variations across configurations. In the end, we selected the model with the best final lacuna IoU value on the validation set (alpha = 0.1), while the small variations observed between the different models (worst = 0.926; best = 0.929) are likely to have limited impact.

### Performance of the full pipeline for estimating the Lacuna to Cortex Ratio

**Fig. 5:**
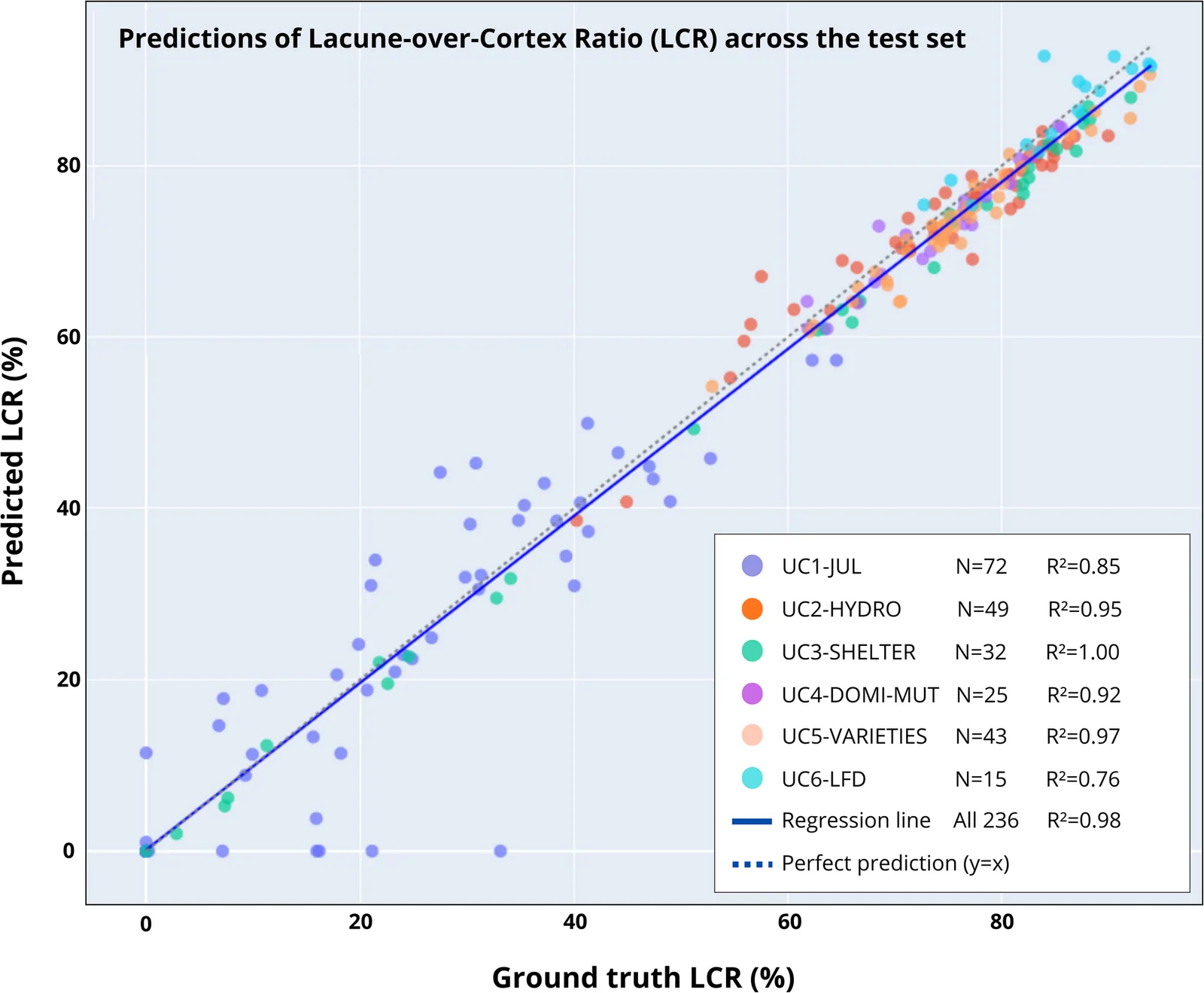
Agreement between predicted and ground truth lacuna-to-cortex ratio. Each point represents one image (*N* = 236), colored by acquisition use-case. The correspondence between predicted measurements (y-axis) and measurements from manual annotation (x-axis) indicates trait quantification performance across heterogeneous datasets.

To evaluate the model’s ability to quantify aerenchyma lacunae and provide these traits for modelling experiments or breeding applications, we analyzed the capacity of the pipeline to predict the target trait of interest (lacuna-to-cortex ratio, LCR), comparing the LCR value obtained with the predicted segmentations with those obtained from manual reference annotations (see Fig. 5). Because breeding relies on trait variation across large populations, we investigated the ability of the model to generalize to unseen data. To that end, we computed the coefficient of determination (R²) between predicted and expected LCR values on the test set images. The coefficient computed on the full test set was very high, indicating a strong agreement with the manual reference (R² = 0.98). We then separated the data by providers (six use cases) and measured the R² within each subset, obtaining consistently high values, which indicated high segmentation performance across use-cases.

Over the six use-cases, two exhibited an R² lower than 0.93. Use-case 6 (UC6-LFD) had the lowest R² value (0.76). However, this result should be interpreted cautiously since the subset contains only 15 images, with a high mean LCR (0.8) and a small standard deviation (0.1). In this context, the range of variations is very small relative to the mean value, making small errors appear disproportionately influential on the R² values.

The second “difficult” use-case (UC1-JUL) had a larger number of images and an LCR range covering most of the overall variability of LCR across the population, while exhibiting a relatively low R² at 0.85, suggesting greater difficulty for the pipeline in this dataset. Visual inspection by independent experts showed that this use case included a high number of young roots, and therefore more early-stage aerenchyma lacunae, which are harder to annotate, and that it was affected by greater annotation ambiguity. This aspect is detailed below, presenting example images and metrics in the results Sect.  3.4, Independent expert review.

### Qualitative assessment of segmentation

**Fig. 6:**
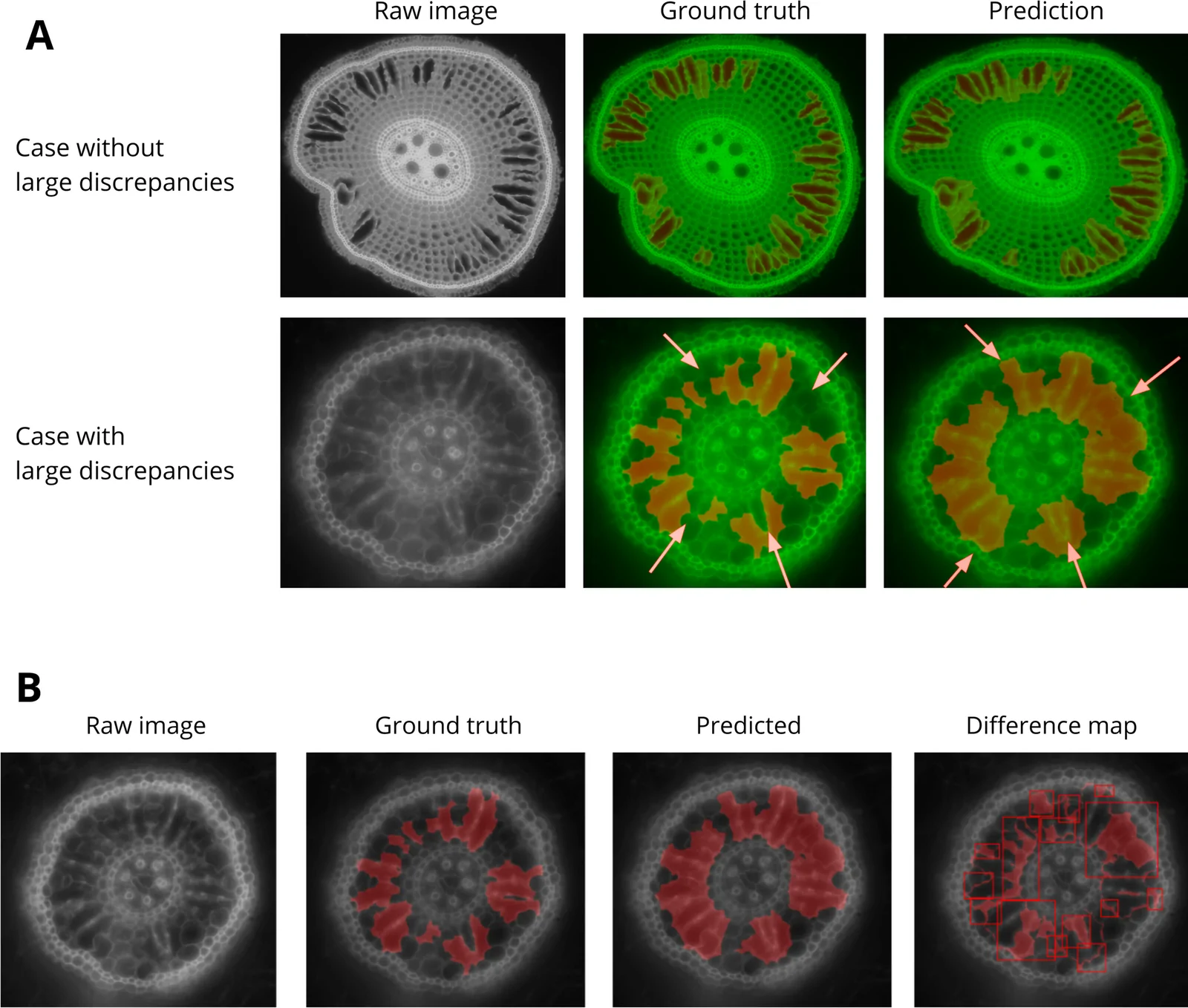
**A**: qualitative examples of automatic segmentation performance on representative root cross-sections. Left: raw image. Middle and right: overlay of raw data (green channel) and segmentation output (red channel). The upper panel shows a high-quality automatic segmentation with accurate identification of aerenchyma lacunae. The lower panel illustrates a challenging case, with large discrepancies between the masks computed from the manual reference annotation and the predicted lacunae. **B**: Expert review of model discrepancies, assisted by an algorithm to highlight the differences.

Visual inspection confirmed that the model reliably captured the shape and position of lacunae across a broad range of rice root cross sections. In clear and well-contrasted images, predicted lacuna masks closely matched ground truth delineations, capturing even small cavities. In more challenging images, especially those with blurred cortical boundaries or uneven cell-wall fluorescence, the model tended to slightly overestimate lacunae shape, but still preserved their general geometry and did not introduce anatomically implausible structures (e.g., lacunae outside the cortex, or scattered parts of lacunae). Occasionally, segmented images displayed substantial predictions errors. In most cases, these unsuccessful cases combined several challenges described earlier (blurry images, low cell wall contrast), with additional difficulties, such as ambiguous lacunae corresponding to young and undifferentiated lacunae, which led to annotation ambiguities (see Fig. 6A).

### Independent expert review

Visual inspections showed that some of the discrepancies between the annotations and the model predictions could be attributable to annotation errors (Fig. 6B). We further investigated this intuition by convening an additional panel of two expert biologists to review a random set of images showing large discrepancies between annotations and predictions (23 images selected among those with the lowest IoU prediction values). Interestingly, a majority of these images were associated with UC1-JUL experimental subset, which had the lowest R² score.

#### Quantitative analysis

We provided the experts with the raw images, masks computed from the manual reference annotations, model predictions, and difference maps (annotations versus predictions). For each mismatch (in the general case, there are multiple mismatches per image), they attributed it to the model, the annotator, or undetermined. These results are presented in Table 2. Across all reviewed images, experts identified 124 annotation errors versus 112 model prediction errors. This indicates a higher number of errors made by annotators, representing an 11% excess relative to model errors. In addition, 16 discrepancies (6% of the differences) could not be attributed, suggesting human perceptual limits or remaining limitations in the problem definition.

#### Qualitative analysis

We then asked the experts to provide a qualitative assessment of the dataset. Importantly, experts noted that manual annotation errors typically consisted of large, misplaced regions with a substantial impact on the computed lacuna percentage, whereas model errors were generally restricted to minor boundary shifts or local contour irregularities. These results support the view that the network at least matches, and sometimes exceeds annotators by avoiding major inconsistencies that likely arise from human subjectivity, objective misunderstanding, or fatigue.

**Table 2:** Results of the expert review of the model errors

Expert	Attribution of discrepancies between annotations and model predictions		
	# Annotation errors	# Model prediction errors	# Undetermined
Expert #1	70	59	3
Expert #2	54	53	13
Total	124	112	16

### Application to rice root phenotyping across environments, genotypes, and growth stages

#### Deployment in biology

The automated segmentation pipeline provides accurate aerenchyma lacunae segmentation while reducing per-image processing time from approximately three minutes per slice (semi-automatic workflow) to approximately two seconds on CPU, and even less on GPU. This gain enables routine analysis of large anatomical datasets, removes operator-dependent variability, and allows breeding teams to quantify aerenchyma lacunae at scales previously inaccessible. The model and codebase have been adopted by collaborating biologists and integrated into ongoing phenotyping campaigns, including the six use cases described in this study.

#### Overview of usage across use cases

The pipeline is currently used across six complementary studies aiming to determine aerenchyma lacuna variability for gene discovery, stress physiology, and breeding-oriented screening. UC1 screens rice mutants to identify genes associated with contrasting cortical aerenchyma lacunae development trajectories. UC2 (Colombia) evaluates a diversity panel of cultivated rice varieties under hydroponics designed to mimic flooded paddy conditions, to characterize anatomical responses to low-oxygen environments. UC3 (Colombia) assesses varietal responses under controlled irrigation regimes under rain-out shelter conditions, quantifying aerenchyma lacunae plasticity under contrasting water supply. UC4 extends UC1 to soil-based growth conditions to test whether mutant phenotypes observed in controlled settings persist in more realistic substrates. Finally, UC5 (France) and UC6 (Colombia) screen diverse germplasm panels to map aerenchyma lacunae profiles and provide trait distributions that can inform upcoming breeding efforts.

#### Focus on an ongoing work in use cases (UC2 and UC3)

**Fig. 7:**
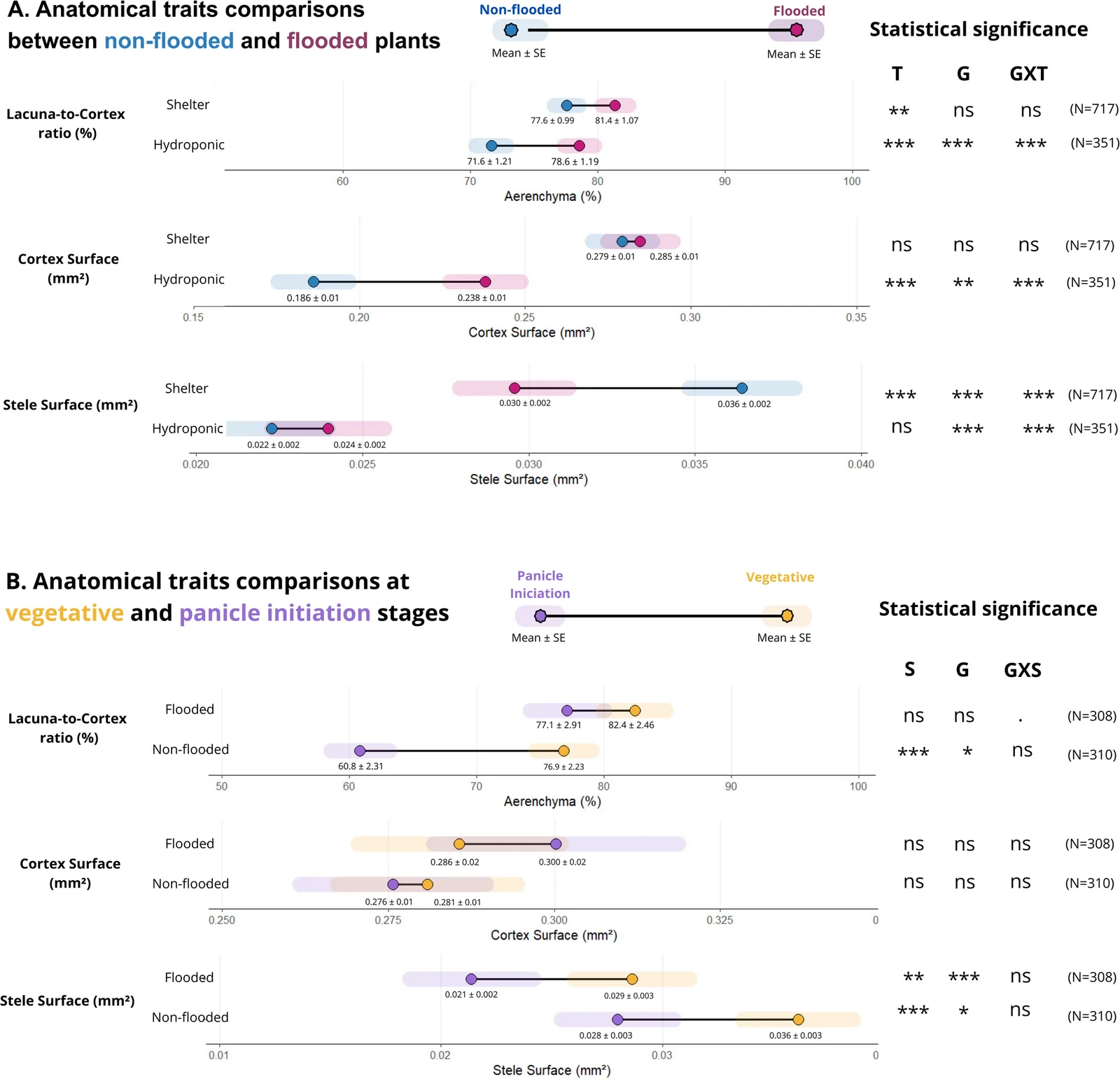
Root anatomical traits variations across environmental and developmental factors in experimental systems with different levels of oxygen availability. Points represent estimated marginal means (EMMs), with horizontal bars indicating ± standard errors, and solid lines represent pairwise contrasts. Statistical significance was assessed using two-sided mixed-model ANOVA, and main effects and interactions are reported on the right side of each panel using standard significance codes: ns, not significant; 0.05 ≤ *P* < 0.10 (.); *P* < 0.05 (* ); *P* < 0.01 (**) and *P* < 0.001 (***). Tested effects include genotype (G), treatment (T), developmental stage (S), and their interactions (GxT, GxS). Each panel reported the sample size (N) corresponding to the number of root cross-section images analyzed per experimental group, obtained from independent plants (biological replicates). **A**: trait comparisons between non-flooded and flooded conditions under field (Shelter) and hydroponic experiment conditions. **B**: trait comparisons between vegetative and panicle initiation developmental stages under contrasting water regimes in field conditions.

Aerenchyma lacunae formation in roots is a key anatomical trait associated with how plants respond to varying levels of soil oxygen availability [18]. Therefore, quantifying root anatomical traits related to aerenchyma lacunae is important for understanding the functional plasticity of the root system in contrasting environments. Root anatomical traits were quantified 1 cm proximal to the root base using the DeepAerenchyma pipeline across multiple experimental contexts (Fig. 7). The lacuna-to-cortex ratio (%) differed significantly between flooded and non-flooded conditions, with significant genotype and genotype x environment effects. Cortex surface area differed between treatments only under hydroponic conditions, whereas stele surface area differed only under soil conditions. Genotypic variation was greater for cortex and stele surface area than for lacuna-to-cortex ratio. For both cortex and stele surface area, genotype and genotype × treatment interactions were significant (Fig. 7A).

To assess the ability of the tool to capture developmental effects on root cortical traits, anatomical variables were compared between the vegetative stage and panicle initiation under shelter conditions (Fig. 7B). Under non-flooded conditions, the proportion of aerenchyma lacunae within the cortex was significantly higher at panicle initiation than at the vegetative stage (*P* < 0.001), whereas no developmental differences were detected under flooded conditions. Cortex surface area did not differ significantly between developmental stages under either water regime (*P* > 0.05). In contrast, stele surface area varied significantly with development under both flooded (*P* < 0.01) and non-flooded (*P* < 0.001) conditions, with stele surface area consistently larger at the vegetative stage. Significant variation in cortex and stele surface area was observed within each treatment and developmental stage, driven by significant genotypic effects.

**Fig. 8:**
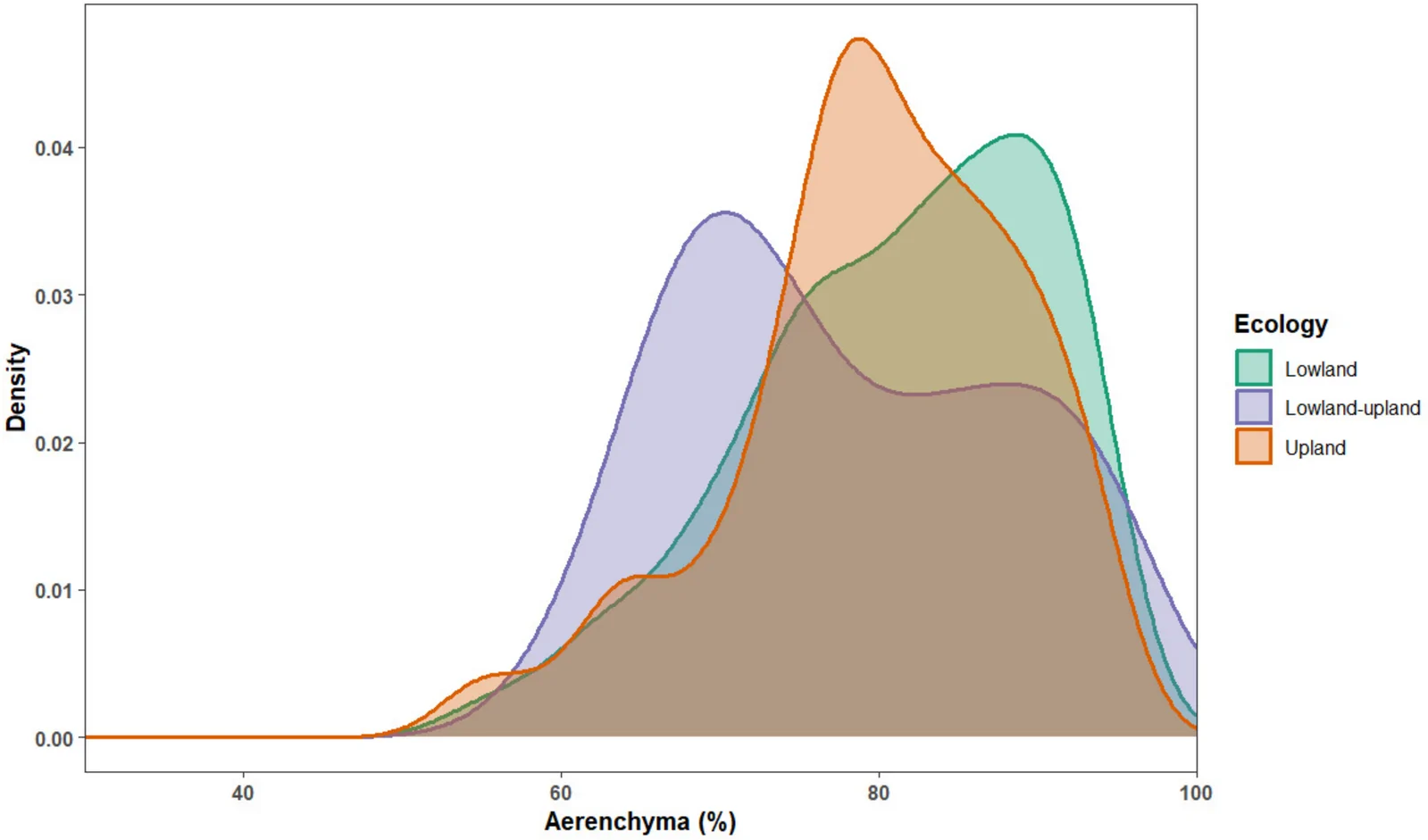
Density distributions of lacuna-to-cortex ratio (%) across ecological groups of rice under aerobic field conditions. Density curves show the frequency distribution of the aerenchyma lacunae values measured in 40 genotypes grouped by ecology (Lowland, Lowland-upland, Upland). Differences in curve position, width, and overlap reflect contrasting ecological variation in root anatomical traits in response to non-flooded growth conditions. Measurements were obtained from independent plants (biological replicates) grown under non-flooded conditions. Sample size (*N* = 600) corresponds to the total number of root cross-section images analyzed.

To evaluate the genotypic variation structure by ecology within a single environment, the lacuna-to-cortex ratio (%) was examined across rice ecological groups under aerobic field conditions (Fig. 8). There were no significant differences in overall mean aerenchyma lacunae in cortex (%) values among ecologies (Type III ANOVA, *P* > 0.05), with no significant contrasts between ecological groups after Tukey adjustment (*P* > 0.05 for all comparisons). Despite this, Lowland, Lowland-upland, and Upland genotypes exhibited partially overlapping distributions, with all ecological groups spanning a wide range (≈ 54–96%). This reflects substantial variability within each ecology under uniform conditions.

Across ecological groups, lacunae-to-cortex (%) exhibited a consistent ordering of mean values, with higher averages in Lowland genotypes (mean ± standard deviation: 81.1 ± 9.34, *N* = 255), intermediate values in Upland genotypes (79.9 ± 10.3, *N* = 300), and lower values in Lowland-upland genotypes (77.9 ± 8.98, *N* = 45). However, density distributions (Fig. 8) revealed that Upland genotypes were characterized by a strong concentration of observations around intermediate values (≈ 70%), whereas Lowland genotypes displayed a distribution shifted toward high values. In this way, the tool demonstrates the variability across genotypes for aerenchyma lacunae formation in the shelter experiment and differences between ecologies.

## Discussion

### A high-throughput, open, and transferable anatomical phenotyping tool

This study demonstrates that a vision transformer architecture enables robust, high-throughput segmentation of rice root anatomy under different experimental and imaging conditions, encompassing variability across countries, research groups, genotypes, growth systems, water regimes, developmental stages, root maturity levels, sampling positions, and acquisition batches. At the imaging level, all images were acquired by epifluorescence microscopy under a harmonized protocol, but across multiple batches, magnifications, and image-quality conditions. By contrast, the validated domain does not include images excluded during curation because of severe blur, truncation, extreme deformation, strong anatomical ambiguity, or the presence of additional structures such as lateral roots that substantially perturb the local cortex organization.

Automated phenotyping of root anatomical traits remains a major bottleneck in root biology and breeding, and existing tools often rely on semi-automatic workflows, site-specific tuning, or restrictive acquisition conditions [2, 3, 7, 14, 24]. By contrast, our SegFormer-based pipeline was trained on a multi-environment dataset encompassing field-derived and controlled images, and did not require any site- or condition-specific calibration to maintain high performance. However, one limitation of our evaluation process: while the test set images are “unseen data” for the model (they were not used for training), these images were acquired along with the images of the training set, during the same experiments. An additional level of validation would be to perform external validation with data from new experiments, and ideally with images from independent research groups. Another limitation of the present work is that the manual reference masks should not be interpreted as an absolute histological ground truth. Because aerenchyma lacunae were not revealed by a lacuna-specific stain, their identification relied on visual morphological criteria. This is particularly challenging in young or weakly differentiated aerenchyma, where partially lysed cortical cells may resemble normal cell lumens, and in advanced aerenchyma development stages where coalescence blurs individual cell boundaries. Consequently, residual annotation ambiguity is expected and was observed in the supplementary examples and in the expert discrepancy review.

In this study, we provided systematic visual results on a representative subsample of the test set, that can be examined in the Additional file 1. To further probe robustness beyond the original study, we performed an additional exploratory qualitative assessment on root cross-section images generated outside our experimental workflow and processed with the same inference pipeline (data not provided). These images covered more heterogeneous and less controlled acquisition conditions, including images with overlaid characters, brightfield microscopy images inverted to match the contrast polarity of fluorescence images, a small maize root cross-section, and a rice root image captured with a smartphone from a microscope control screen and submitted through the Hugging Face interface over a 3G connection. Across these examples, the outputs remained qualitatively coherent and anatomically plausible, despite occasional local errors in cortex or lacuna estimation. Because these data were not accompanied by pixel-level annotations, they were not used for quantitative interpretation and should not be considered a formal validation; however, they provide preliminary indications of potential robustness beyond the training domain.

The present study demonstrates robustness across a broad range of rice root cross-section images. It was trained on seminal and crown root sections sampled at several positions and maturity levels, which provides some tolerance to variation in sampling location. However, the current validation does not cover all rice root classes, such as lateral roots or very young tip sections, and does not extend formally to non-rice species with similar anatomy. Although the dataset includes multiple rice genotypes, growth systems, developmental stages, sampling positions, acquisition batches, and two institutions, it does not constitute a formal external validation on images produced independently by laboratories outside this study.

The model should not be considered validated for maize or for other non-rice species, even when the general root anatomy appears superficially similar. In out-of-distribution images, anatomical differences in stele organization, xylem vessel appearance, root size, or cortical structure may induce specific failure modes. Transfer to maize or other species would require a dedicated annotated dataset, species-specific evaluation, and possibly model fine-tuning. Accordingly, extension to other species should be approached with caution.

The combination of a lightweight decoder and a resolution-agnostic transformer encoder enabled efficient inference while preserving anatomical detail, making the method compatible with large-scale deployment and CPU-accessible usage. This design choice supports practical large-scale phenotyping workflows, where thousands of images from multiple sources must be processed rapidly and reproducibly. The open release of code, trained models, annotation guidelines, and an interactive online demonstration ensures transparency, reproducibility, and reusability, aligning with current recommendations for artificial intelligence-driven plant phenotyping tools [19, 23]. In practical terms, the pipeline’s scalability has already been demonstrated by its deployment in ongoing phenotyping campaigns, processing thousands of images from multiple field sites, with minimal additional human intervention.

### Expert-level precision and reduced subjectivity relative to manual annotation

Beyond throughput, our results highlight a critical advantage of deep learning for root aerenchyma lacuna phenotyping: increased consistency relative to human annotations. While expert manual segmentation remains the reference standard, it is inherently limited by subjectivity, fatigue, and ambiguity in class boundaries, particularly for small or developing aerenchyma lacunae. Annotation variability is well documented in bioimage analysis and can substantially hinder both model training and biological interpretation [19, 22].

In our study, an independent expert audit showed that model predictions matched or exceeded the consistency of individual human annotators. Notably, the model handled challenging cases with greater consistency than individual human annotators. Model errors were primarily limited to subtle boundary shifts, whereas manual annotations occasionally exhibited larger inconsistencies, especially in datasets dominated by young roots with small or poorly differentiated lacunae. We interpret this behavior as a consequence of training on pooled expert knowledge across datasets, which promotes anatomical coherence and reduces individual bias. Similarly, in biomedical imaging, multi-expert learning frameworks such as GroupLearning [5] show that models trained on annotations from multiple experts can mitigate individual rater bias and yield more stable and reproducible predictions for tasks affected by perceptual ambiguity.

However, our results suggest that further improvement in aerenchyma lacuna segmentation accuracy could be achieved by: (i) improving annotator guidance for young lacuna, for example by refining the annotation guide and conducting early consensus reviews on a subset of annotated images; (ii) increasing the number of annotated images, as the current training set relies heavily on data augmentation (random rotations, translations and mirror operations from approximately 1,700 original images); and (iii) building on these improvements, by exploring additional training configurations, larger architectures than SegFormer-B4, potentially at the expense of deployability, or redesigning the training workflow using annotation-efficient approaches such as active learning.

### Discrimination of root cortical traits across genotypes, environments, and developmental stages

Automated segmentation translated into biologically meaningful trait quantification. The AI-based framework consistently discriminated genotypic effects, environmental responses, and developmental shifts in cortex and stele surface areas as well as lacuna-to-cortex ratios across hydroponic and soil-based systems. These results demonstrate that automated image analysis captured established anatomical responses while revealing fine-scale variation across contexts.

Increased aerenchyma lacunae formation under flooded or oxygen-deficient conditions was detected in both hydroponic and shelter experiments, consistent with established physiological mechanisms driven primarily by oxygen availability rather than soil properties per se [8, 13, 18]. Differences observed between hydroponic and soil-grown roots further suggest that mechanical impedance influences of mechanical impedance on cortical and stele development, in line with previous reports on root anatomical plasticity [11, 16, 17]. The tool also captured developmental effects, including shifts in aerenchyma lacunae proportion and stele size between vegetative and reproductive stages, demonstrating its sensitivity to growth-related anatomical dynamics [6, 11].

## Conclusion

This study demonstrates that transformer-based image analysis enables robust, scalable, and reproducible quantification of cortical aerenchyma lacunae across heterogeneous experimental conditions. By supporting consistent trait extraction across genotypes, environments, and developmental stages, the proposed pipeline addresses a major bottleneck in rice root anatomical phenotyping.

The ability to capture large genotypic variation in cortical traits highlights the potential of artificial intelligence-enabled phenotyping for breeding and diversity studies. By providing high-throughput, transferable anatomical measurements, this framework facilitates the integration of rice root structural traits into physiological modelling, genetic analyses, and genomic selection strategies, and supports systematic investigation of root anatomical plasticity and contributes to the development of climate-smart rice improvement programs.

## Supplementary Information

Below is the link to the electronic supplementary material.

Supplementary Material 1.

## Data Availability

The multi-environment multi-site dataset used for training is available upon reasonable request, subject to data-sharing agreements. To ensure reproducibility by the community, all code used for preprocessing and training is released under an open-source licence on GitHub, tagged v1.0.2, and archived with a Zenodo DOI (Atef, 2025). For users who want to run the pipeline on images, two convenient options are provided with the HuggingFace space (Fernandez and Atef, 2025): (I) an interactive web demonstrator deployed as Hugging Face Space, which requires no installation and includes a subset of the test dataset; or (ii) a local version of the pipeline available from the same repository, which can be cloned from the tab “Files” of the space, installed following the instructions provided in the README.md, by installing the libraries defined by the requirements, and executing the run_locally.py script on any machine equipped with a GPU and a standard Python environment.The online demonstrator allows non-specialists to upload a batch of images stored in a ZIP archive and obtain predictions in a limited time (approximately 2 s per image) without any local installation. The offline script can process images at a significantly faster throughput (0,08 s per image, as measured on a recent lenovo laptop with a nvidia GPU). Project name: DeepAerenchyma. Project home page (accessed on 6th april 2026): https://huggingface.co/spaces/RomainFernandez/DeepAerenchyma. Archived version of code for preprocessing and training: https://doi.org/10.5281/zenodo.17917313. Operating system(s): Platform independent. Programming language: Python. License: MIT License. Any restrictions to use by non-academics: None.
