# Supplementary material for: Deep aerenchyma: a transformer-based pipeline for scalable phenotyping of rice root aerenchyma lacunae across environments
Source: Plant Methods. 2026 Jun 30;22:83. doi: 10.1186/s13007-026-01546-1 (PMC13595614; doi:10.1186/s13007-026-01546-1)

Additional File 1.
Demonstration dataset of the Hugging Face Space with a comparison of the annotation and the segmentation outputs

This supplementary material provides the complete set of examples included in the online Hugging Face demo dataset. Each example is presented with (i) the raw input image, (ii) the target masks derived from the manual annotations and used for training, called “ground truth”, (iii) the segmentation predicted by the model, and (iv) a difference map comparing prediction and target. In the difference maps, white indicates lacunae correctly identified by the model, blue indicates annotated lacunae missed by the model, and red indicates regions predicted as lacunae by the model but not present in the annotation mask.

This dataset provide a balanced set (5 images for each use case), combining the “easiest cases” (UC2, with almost 100 % of the cortex being lacunae, with the “hardest ones” (UC1, with early aerenchyma, sometimes challenging to identify). The results include examples with strong agreements between annotators and the model, and other examples with small or major discrepancies, sometimes attributable to the model, or to the annotator.

UC1-JULI-01/M2P1_T356_3.4_3cm/3.2.tif

Raw | **Ground truth** | **Predicted** | **Differences**

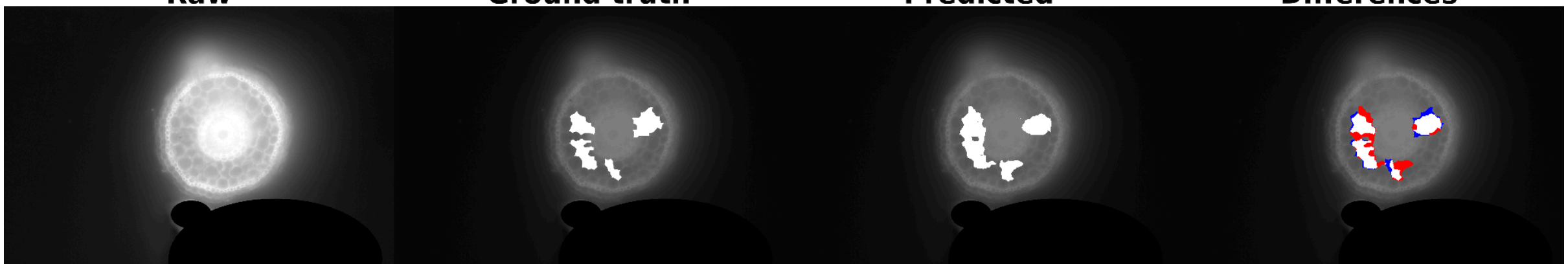

UC1-JULI-01/M2P1_T356_3.4_3cm/3.3.tif

Raw | **Ground truth** | **Predicted** | **Differences**

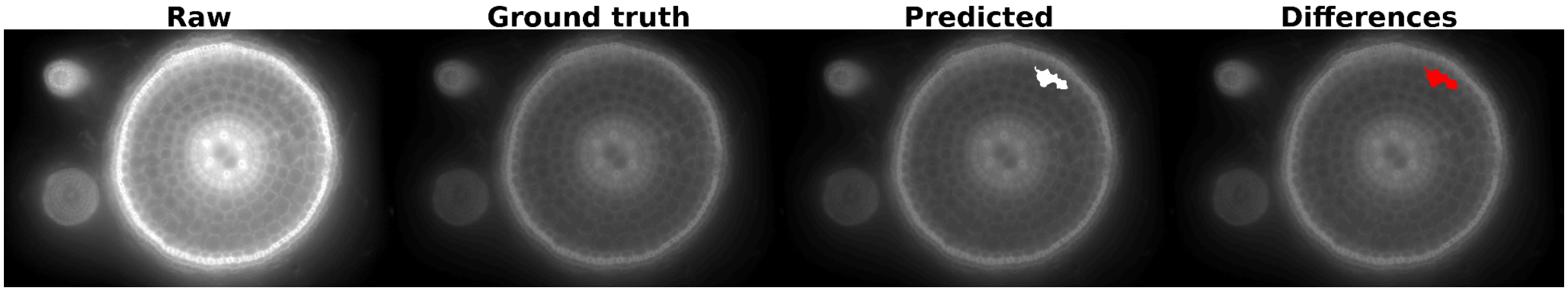

UC1-JULI-01/M2P1_T356_3.4_3cm/3.6.tif

Raw | **Ground truth** | **Predicted** | **Differences**

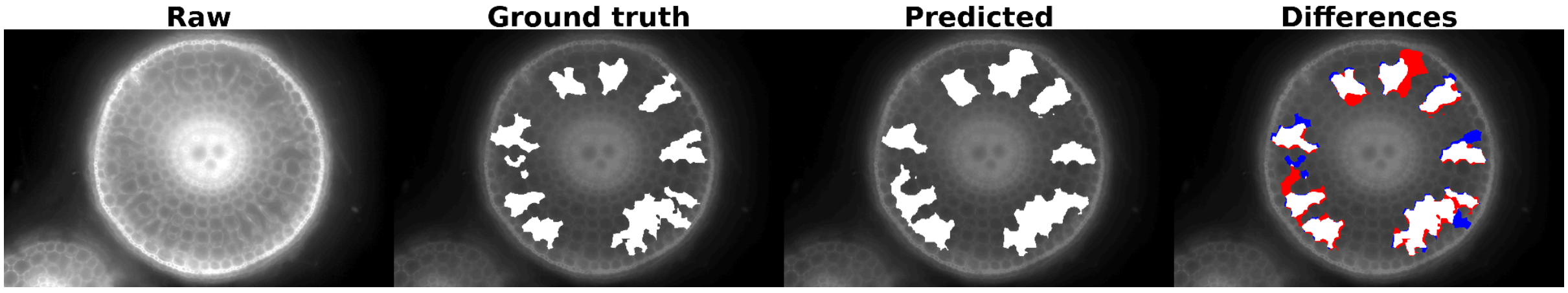

UC1-JULI-01/M2P1_T356_3.4_3cm/3.7.tif

Raw | **Ground truth** | **Predicted** | **Differences**

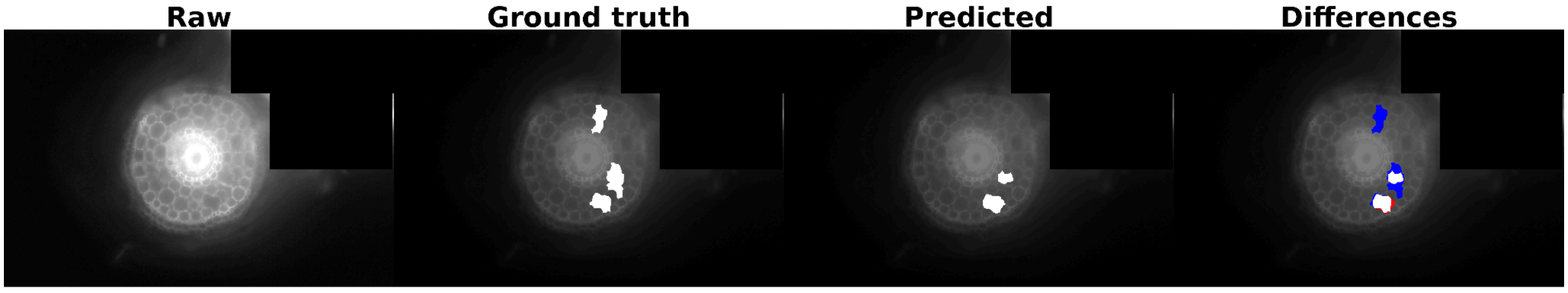

UC1-JULI-01/M2P1_T356_3.4_3cm/3.8.tif

Raw | **Ground truth** | **Predicted** | **Differences**

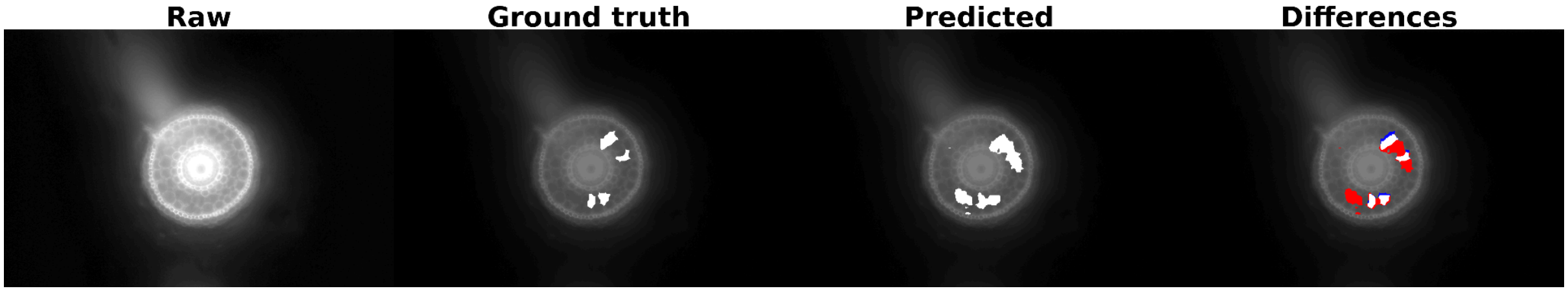

UC2-HYDROPONICS/G16/16_A1SB_1.tif

Raw | Ground truth | Predicted | Differences

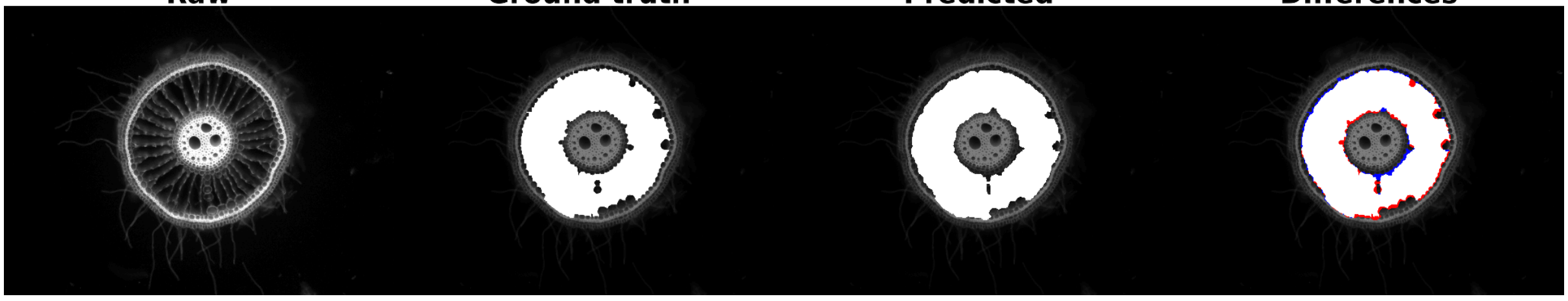

UC2-HYDROPONICS/G16/16_A1SB_2.tif

Raw | Ground truth | Predicted | Differences

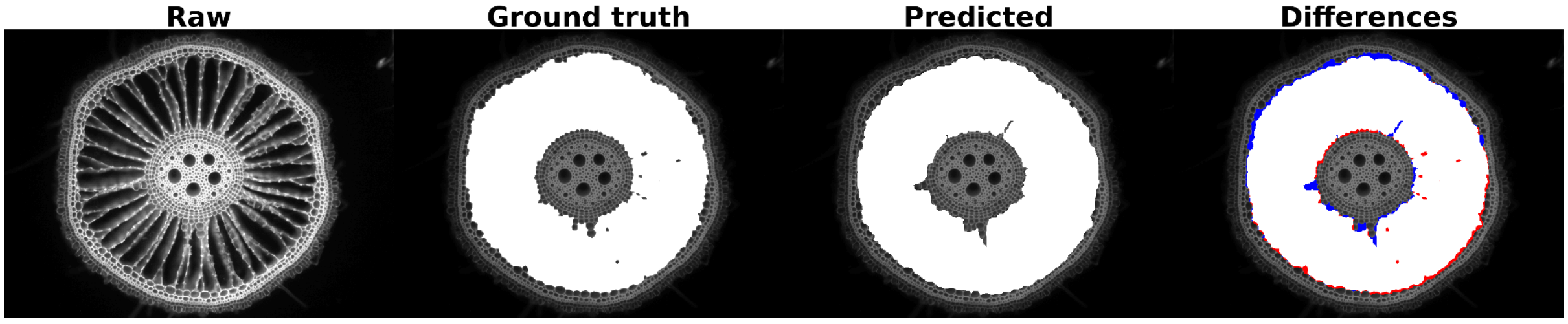

UC2-HYDROPONICS/G16/16_H1SB_1.tif

Raw | Ground truth | Predicted | Differences

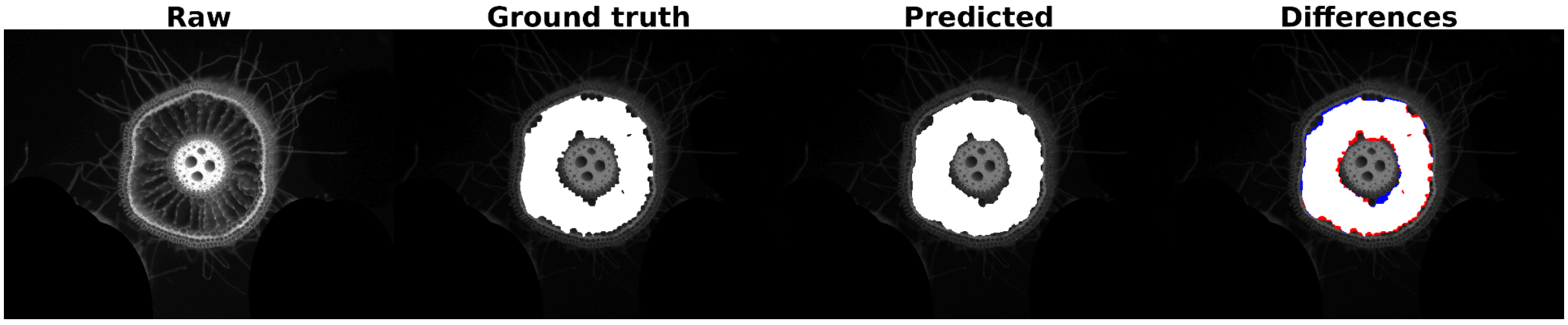

UC2-HYDROPONICS/G16/16_H1SB_2.tif

Raw | Ground truth | Predicted | Differences

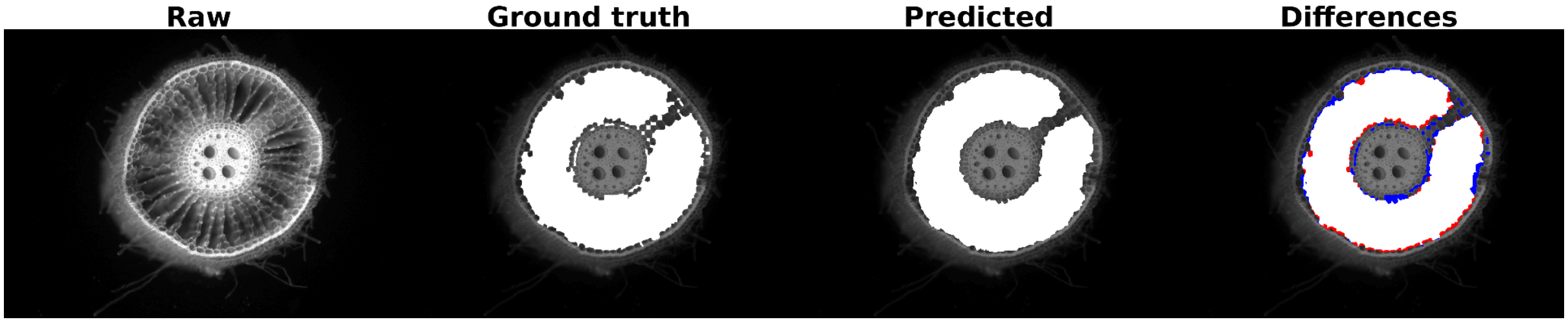

UC2-HYDROPONICS/G16/16_H1SB_3.tif

Raw | Ground truth | Predicted | Differences

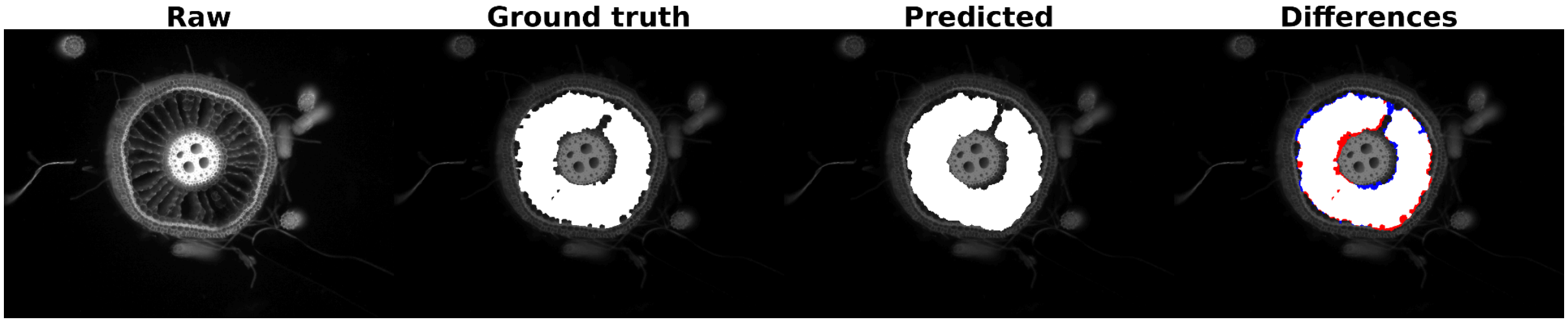

UC2-Shelter/Bloque_A-3_IP_PRIORITARIOS/8_A1SB_2.tif

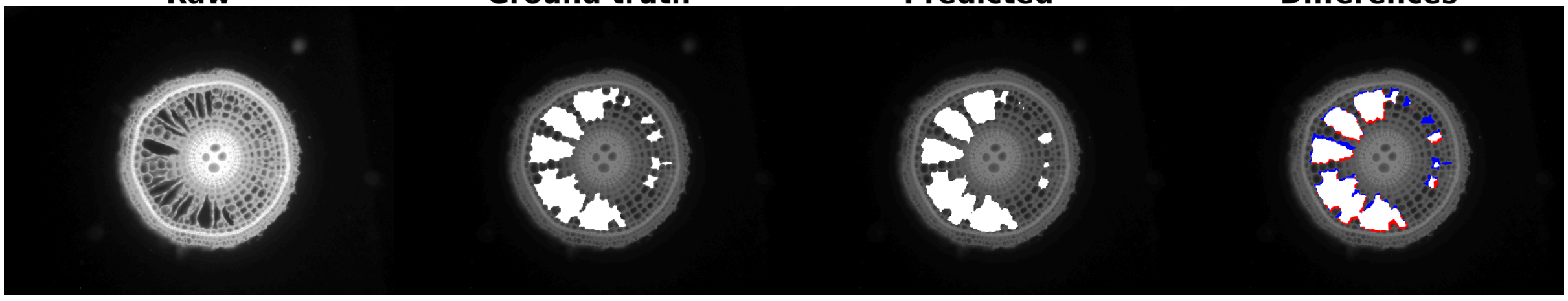

UC2-Shelter/Bloque_A-3_IP_PRIORITARIOS/14_A1SB_1.tif

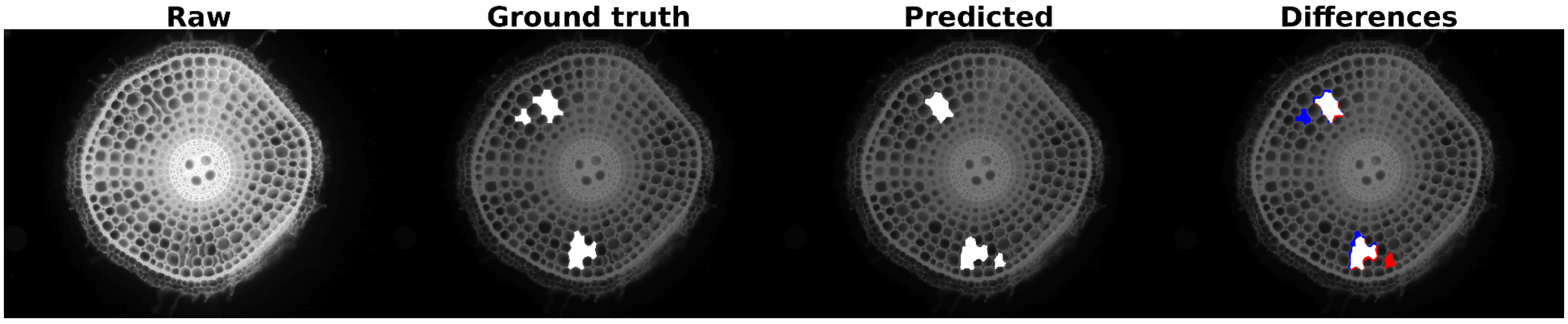

UC2-Shelter/Bloque_A-3_IP_PRIORITARIOS/14_A1SB_2.tif

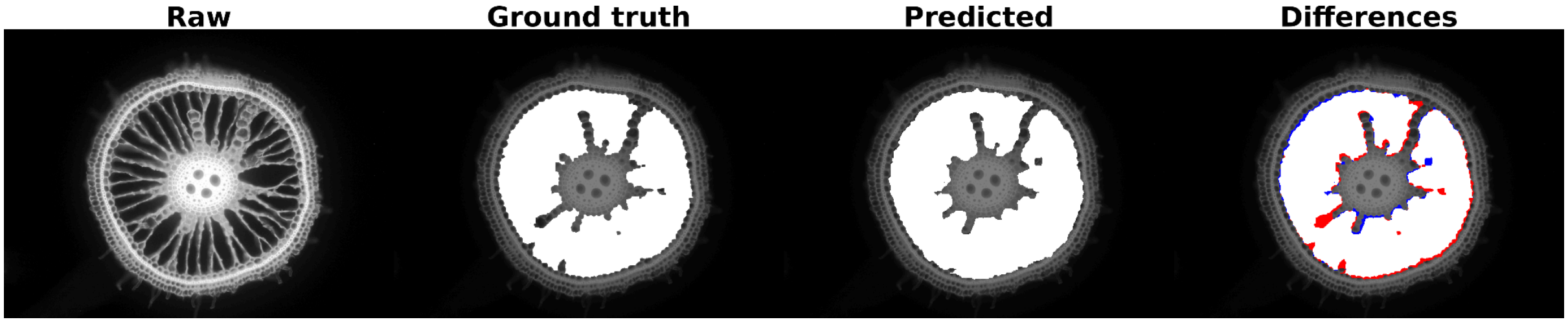

UC2-Shelter/Bloque_A-3_IP_PRIORITARIOS/16_A1SB_1.tif

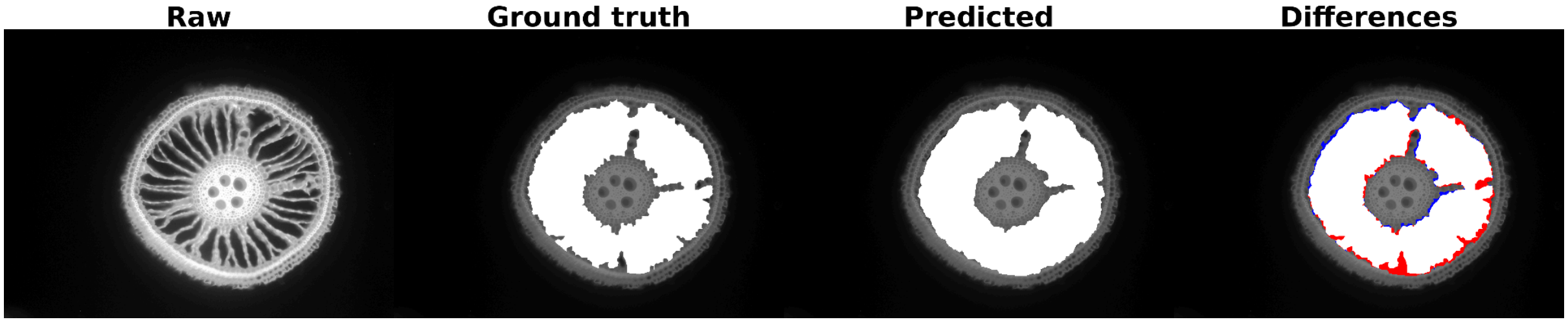

UC2-Shelter/Bloque_A-3_IP_PRIORITARIOS/16_A1SB_3.tif

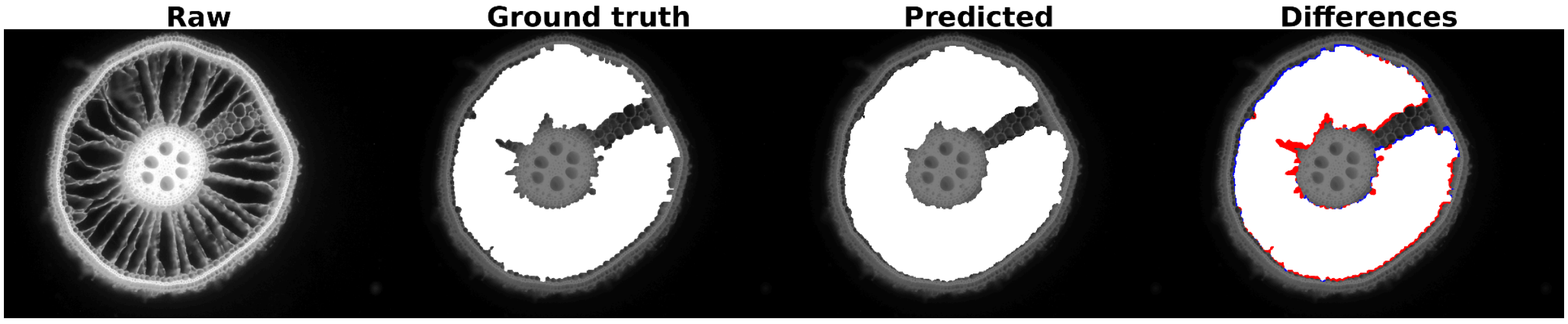

UC3-DOMI-MUT/processing_rep_3/R31_a.tif

Raw | Ground truth | Predicted | Differences

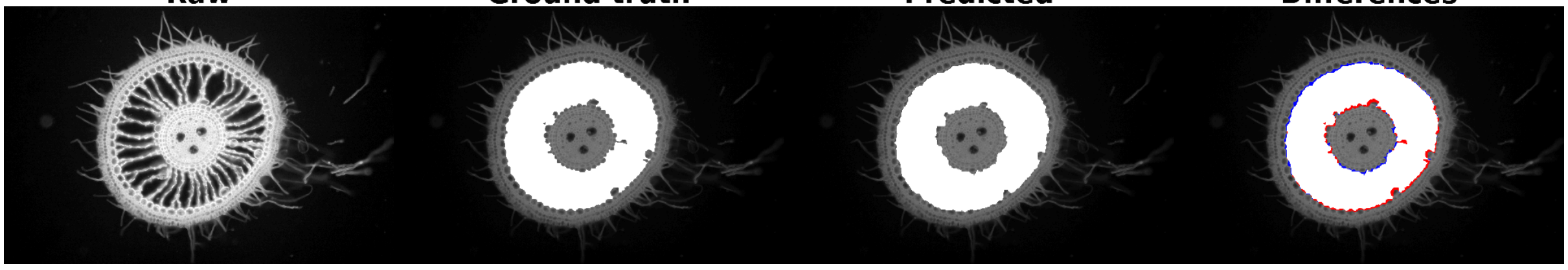

UC3-DOMI-MUT/processing_rep_3/R33_a.tif

Raw | Ground truth | Predicted | Differences

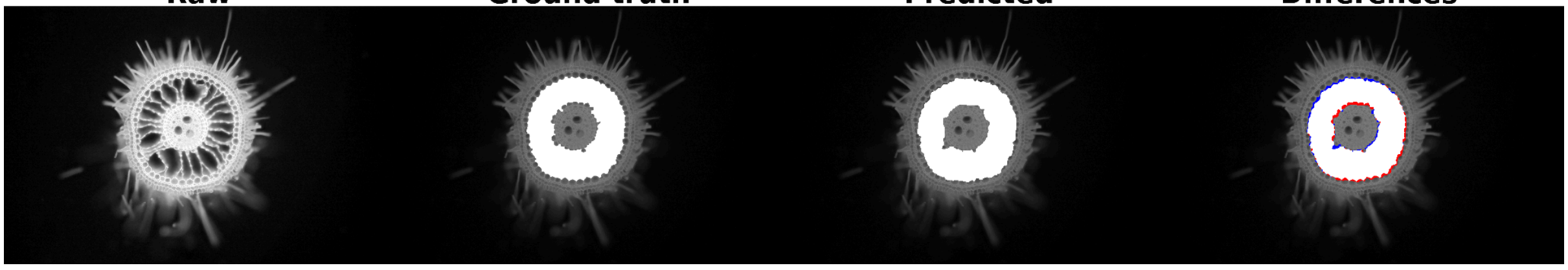

UC3-DOMI-MUT/processing_rep_3/R34_a.tif

Raw | Ground truth | Predicted | Differences

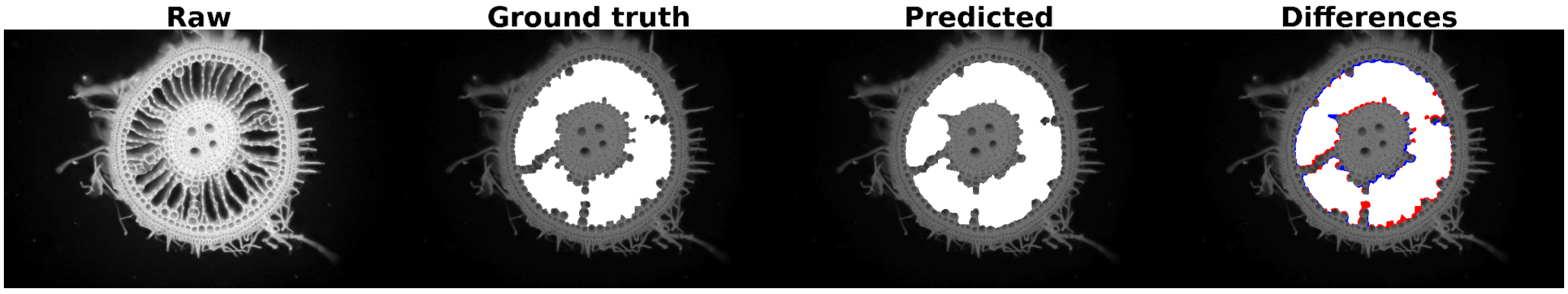

UC3-DOMI-MUT/processing_rep_3/R36_a.tif

Raw | Ground truth | Predicted | Differences

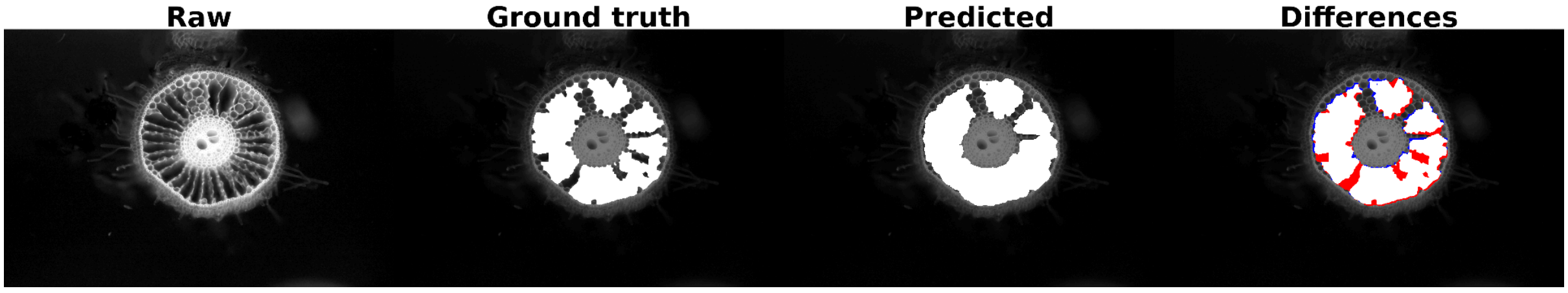

UC3-DOMI-MUT/processing_rep_3/R38_a.tif

Raw | Ground truth | Predicted | Differences

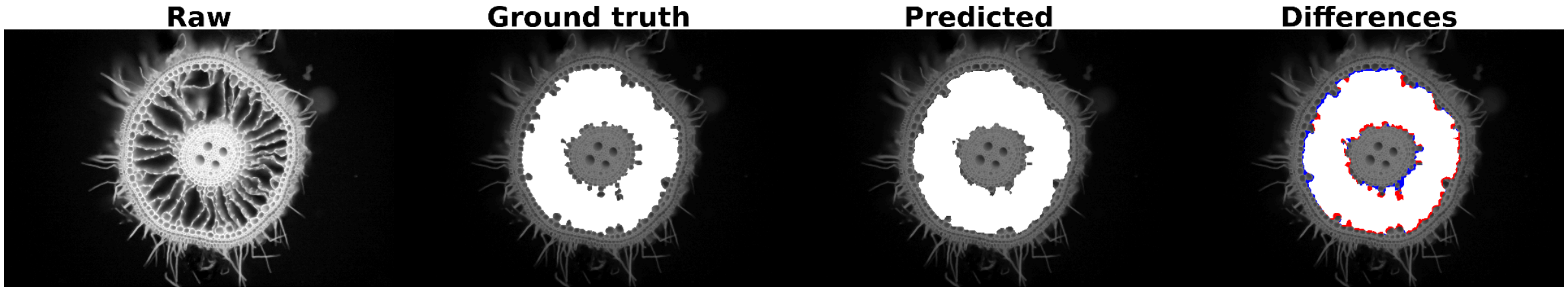

UC3-DOMI-VARI/processing_rep_4/R100_a.tif

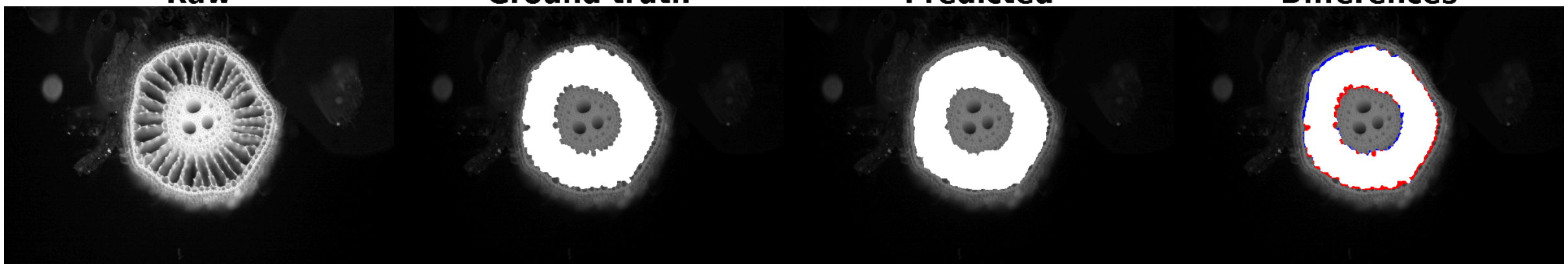

UC3-DOMI-VARI/processing_rep_4/R101_a.tif

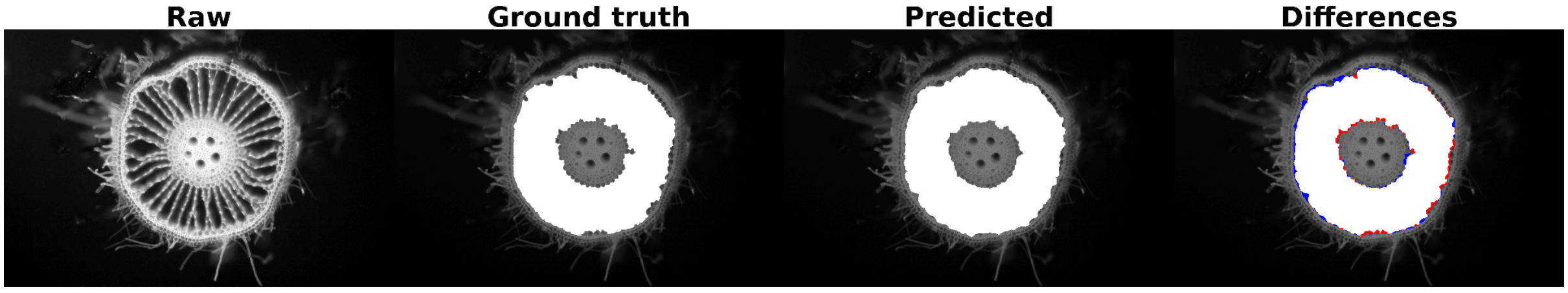

UC3-DOMI-VARI/processing_rep_4/R103_a.tif

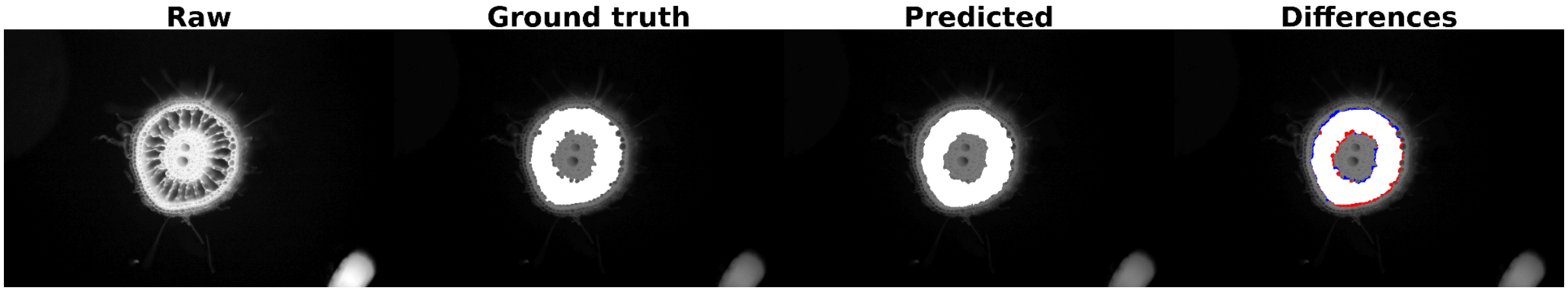

UC3-DOMI-VARI/processing_rep_4/R104_a.tif

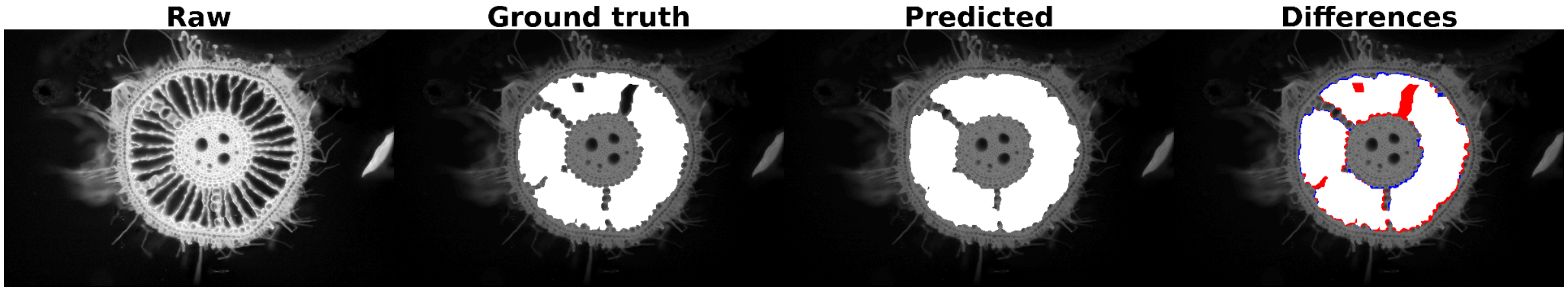

UC3-DOMI-VARI/processing_rep_4/R105_a.tif

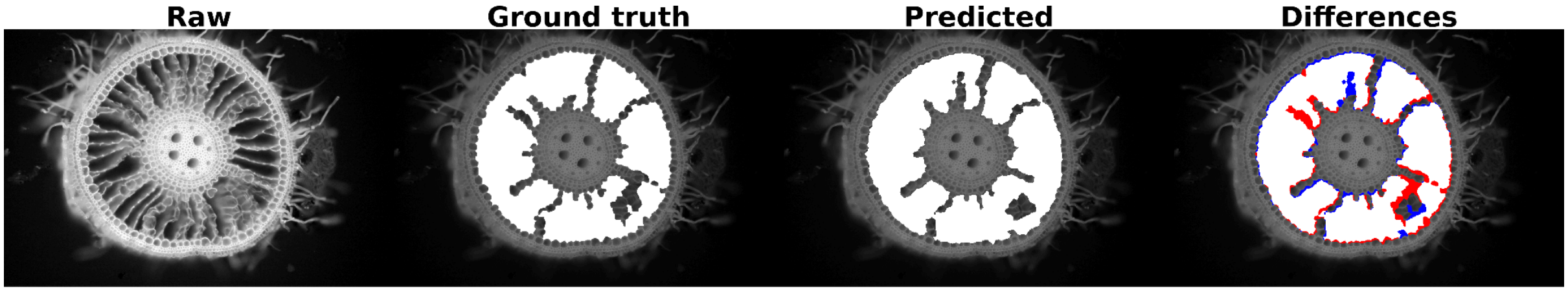

UC3-LAURA_VARI/Rep2_EXP_PROCESS/20_R1_a.tif

Raw | **Ground truth** | **Predicted** | **Differences**

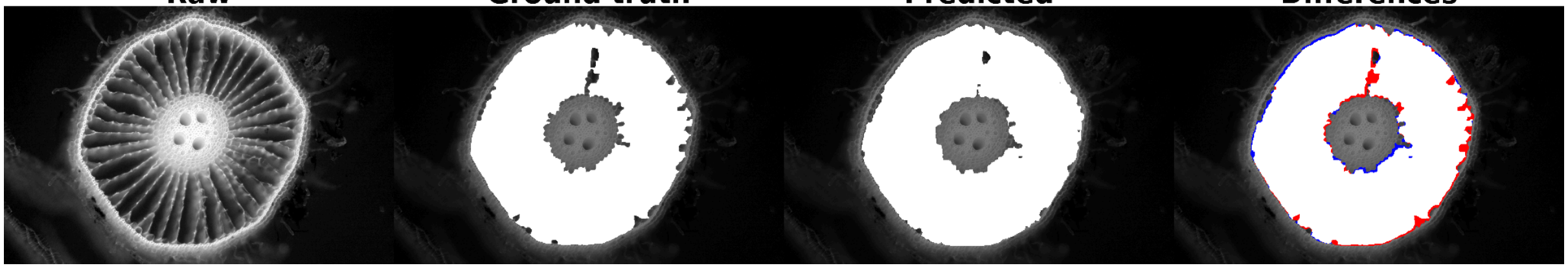

UC3-LAURA_VARI/Rep2_EXP_PROCESS/20_R1_b_bf.tif

Raw | **Ground truth** | **Predicted** | **Differences**

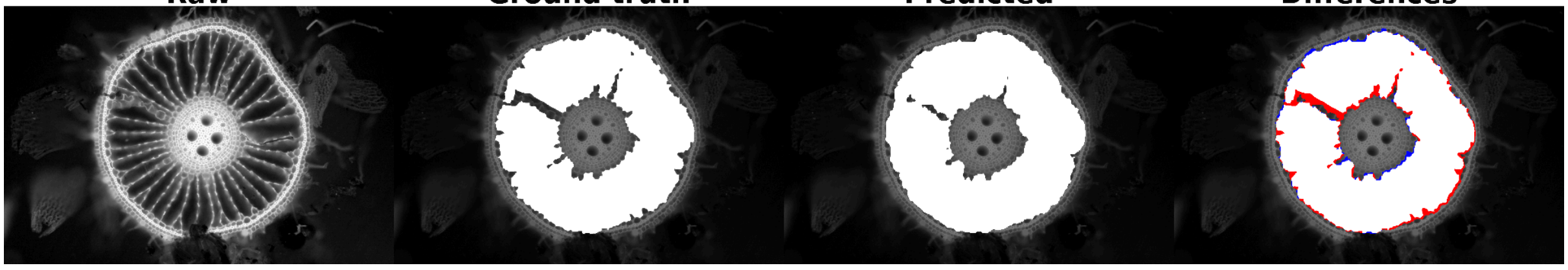

UC3-LAURA_VARI/Rep2_EXP_PROCESS/20_R1_c_bf.tif

Raw | **Ground truth** | **Predicted** | **Differences**

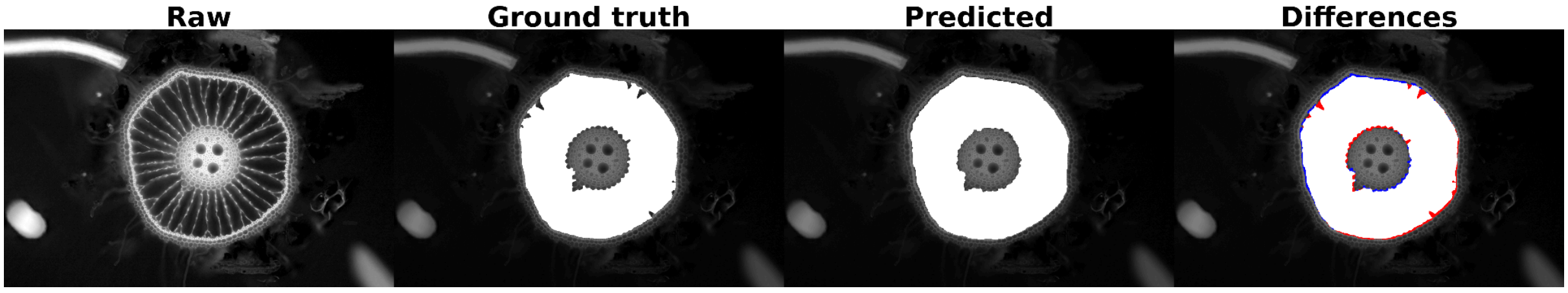

UC3-LAURA_VARI/Rep2_EXP_PROCESS/21_R1_b_bf.tif

Raw | **Ground truth** | **Predicted** | **Differences**

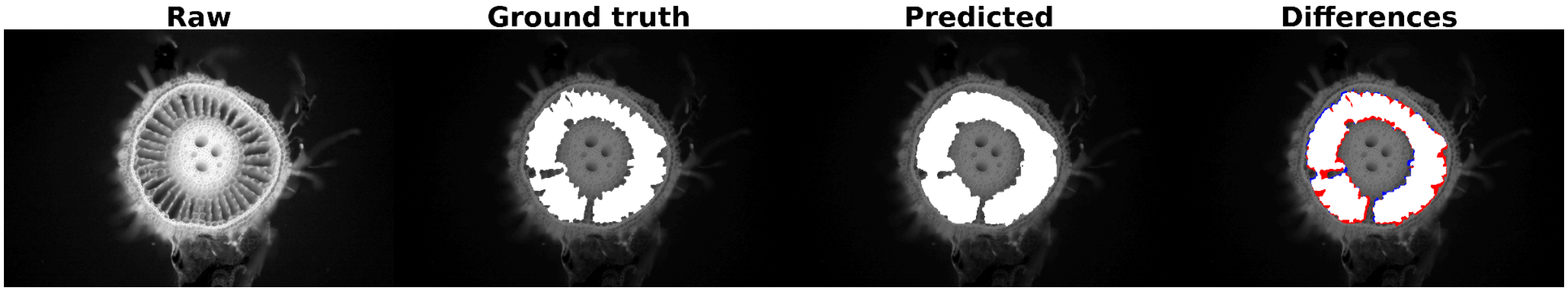

UC3-LAURA_VARI/Rep2_EXP_PROCESS/21_R1_c.tif

Raw | **Ground truth** | **Predicted** | **Differences**

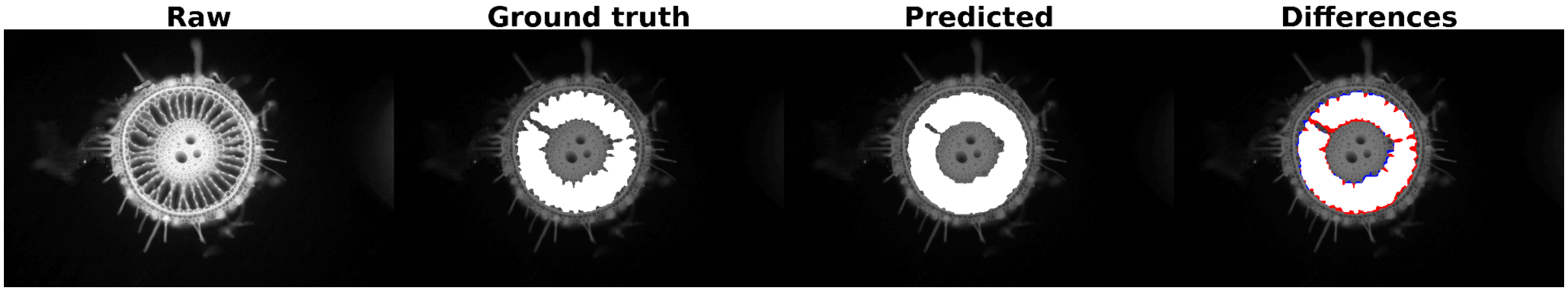

Supplement: Supplementary file 1 [file 13007_2026_1546_MOESM1_ESM.pdf]
